# Buqi-Huoxue-Tongnao decoction drives gut microbiota-derived indole lactic acid to attenuate ischemic stroke via the gut-brain axis

**DOI:** 10.1186/s13020-024-00991-1

**Published:** 2024-09-15

**Authors:** Yarui Liu, Peng Zhao, Zheng Cai, Peishi He, Jiahan Wang, Haoqing He, Zhibo Zhu, Xiaowen Guo, Ke Ma, Kang Peng, Jie Zhao

**Affiliations:** 1 Guangdong Provincial Key Laboratory of New Drug Screening, NMPA Key Laboratory for Research and Evaluation of Drug Metabolism and Guangdong-Hong Kong-Macao Joint Laboratory for New Drug Screening, School of Pharmaceutical Sciences, Southern Medical University, Guangzhou, 510515 Guangdong China; 2 Peng Kang National Famous Traditional Chinese Medicine Expert Inheritance Studio, Integrated Hospital of Traditional Chinese Medicine, Southern Medical University, Guangzhou, 510315 Guangdong China; 3 Integrated Hospital of Traditional Chinese Medicine, Southern Medical University, Guangzhou, 510315 Guangdong China; 4 Microbiome Medicine Center, Department of Laboratory Medicine, Zhujiang Hospital, Southern Medical University, Guangzhou, 510280 China

**Keywords:** Ischemic stroke, Buqi-Huoxue-Tongnao decoction (BHTD), Gut microbiota, Gut-brain axis, Tryptophan metabolism, Indole lactic acid

## Abstract

**Background:**

Ischemic stroke belongs to “apoplexy” and its pathogenesis is characterized by qi deficiency and blood stasis combining with phlegm-damp clouding orifices. Buqi-Huoxue-Tongnao decoction (BHTD) is a traditional Chinese medicine formula for qi deficiency, blood stasis and phlegm obstruction syndrome. However, its efficacy and potential mechanism on ischemic stroke are still unclear. This study aims to investigate the protective effect and potential mechanism of BHTD against ischemic stroke.

**Materials and methods:**

Middle cerebral artery occlusion (MCAO) surgery was carried out to establish an ischemic stroke model in rats. Subsequently, the rats were gavaged with different doses of BHTD (2.59, 5.175, 10.35 g/kg) for 14 days. The protective effects of BHTD on the brain and gut were evaluated by neurological function scores, cerebral infarction area, levels of brain injury markers (S-100B, NGB), indicators of gut permeability (FD-4) and bacterial translocation (DAO, LPS, D-lactate), and tight junction proteins (Occludin, Claudin-1, ZO-1) in brain and colon. 16S rRNA gene sequencing and metabolomic analysis were utilized to analyze the effects on gut microecology and screen for marker metabolites to explore potential mechanisms of BHTD protection against ischemic stroke.

**Results:**

BHTD could effectively mitigate brain impairment, including reducing neurological damage, decreasing cerebral infarction and repairing the blood–brain barrier, and BHTD showed the best effect at the dose of 10.35 g/kg. Moreover, BHTD reversed gut injury induced by ischemic stroke, as evidenced by decreased intestinal permeability, reduced intestinal bacterial translocation, and enhanced intestinal barrier integrity. In addition, BHTD rescued gut microbiota dysbiosis by increasing the abundance of beneficial bacteria, including *Turicibacter* and *Faecalibaculum*. Transplantation of the gut microbiota remodeled by BHTD into ischemic stroke rats recapitulated the protective effects of BHTD. Especially, BHTD upregulated tryptophan metabolism, which promoted gut microbiota to produce more indole lactic acid (ILA). Notably, supplementation with ILA by gavage could alleviate stroke injury, which suggested that driving the production of ILA in the gut might be a novel treatment for ischemic stroke.

**Conclusion:**

BHTD could increase gut microbiota-derived indole lactic acid to attenuate ischemic stroke via the gut-brain axis. Our current finding provides evidence that traditional Chinese medicine can ameliorate central diseases through regulating the gut microbiology.

**Supplementary Information:**

The online version contains supplementary material available at 10.1186/s13020-024-00991-1.

## Background

Ischemic stroke (IS) is caused by the narrowing or occlusion of cerebral blood supply arteries. When cerebral blood flow is interrupted, ATP synthesis is disrupted due to oxygen and glucose deficiency [1]. Further, the dysfunction of ion channels leads to calcium overload, pro-inflammatory factor expression and free oxygen radical production [2, 3]. These changes eventually cause cell necrosis and neurological dysfunction [4]. Stroke belongs to “apoplexy” in the traditional Chinese medicine (TCM) theory, and its pathogenesis is characterized by qi deficiency and blood stasis combining with phlegm-damp clouding orifices [5]. Thus, “invigorating qi,” “activating blood” and “dissipating phlegm” in combination are the basic therapies of apoplexy [6]. Therefore, prescriptions composed of herbal medicines with corresponding functions are often applied in TCM clinics.

Buqi-Huoxue-Tongnao decoction (BHTD) is prescribed by Professor Kang Peng, a nationally prestigious Chinese physician, and exerts the efficiency of “invigorating qi and promoting blood circulation, dissipating phlegm and dredging brain.” BHTD consists of ten-flavored herbal medicines following the compatibility principle of “Monarch-Minister-Assistant-Guide” (Jun-Chen-Zuo-Shi) (see Table S1 for details) [7]. Astragali Radix and Chuanxiong Rhizoma are the Monarchs, which dominate the therapeutic effects targeting the cause of the disease or the main symptom. Both of them and their bioactive ingredients (Astragaloside IV and Z-Ligustilide) have been proven to have neuroprotective effects [8, 9]. Salviae Miltiorrhizae Radix et Rhizoma, Notoginseng Radix Et Rhizoma, Paeoniae Radix Rubra and Pheretima are the Ministers. All four herbs or their extracts have anti-inflammatory activities and attenuate cerebral ischemic injury [10–13]. Poria, Puerariae Lobatae Radix and Pinelliae Rhizoma Praeparatum are the Assistants, in which Puerariae Lobatae Radix is rich in flavonoids that can improve cerebral microcirculation [14, 15]. Glycyrrhizae Radix Et Rhizoma Praeparata Cum Melle is the Guide and can harmonize all of the herbs. BHTD has been demonstrated to have favorable efficacy against cerebral circulatory insufficiency [16]. However, its protective efficacy and mechanism in ischemic stroke remain to be elucidated, necessitating further investigation.

TCM emphasizes the concept of holism, indicating that the internal organs of the human body are inseparable and interconnected, such as “the lung and the large intestine are interior-exterior” and “heart governs mind” [17–19]. Clinically, approximately 50% of patients with apoplexy have gastrointestinal complications, such as gut microbiota dysbiosis, constipation and gastrointestinal bleeding, who have a longer hospital stay, poorer prognoses, and even higher mortality rates [20–22]. This indicates a connection between the gut and the brain. Xu et al. have proved that gut microbiome dysbiosis, represented by *Enterobacteriaceae* overgrowth, would exacerbate brain infarction [23]. Ingestion of multi-strain *Lactobacillus* and *Bifidobacterium* has been confirmed to improve neurological deficits by revamping intestinal integrity [24]. This indicates the gut microbiota can mediate the crosstalk between the digestive system and the central nervous system, known as the “gut-brain axis” [25, 26]. Furthermore, metabolites derived from the gut microbiota can be absorbed into the blood circulation by intestinal epithelial cells, thus affecting the physiological functions as well as the metabolism of the host [27]. A clinical cohort study indicated that the activity of tryptophan metabolism in the kynurenine pathway is positively connected with the degree of stroke and the prognosis over the long term [28]. In previous research, we have demonstrated that melatonin, a tryptophan metabolite in the serotonin pathway of the gut microbiota, could enhance intestinal barrier function to attenuate brain injury [29]. These demonstrate that tryptophan metabolism in the gut is critical for the treatment of ischemic stroke. Indoles, tryptophan metabolites via the gut microbiota, are ligands for aryl hydrocarbon receptor (AHR), which have been confirmed to maintain the epithelial barrier [30]. For example, indole lactic acid (ILA), with the function of ameliorating intestinal inflammation, can significantly enhance neurite growth in PC12 cells [31, 32]. Therefore, indoles have great potential for regulating the gut-brain axis. In this study, we investigated the protective effects of BHTD against ischemic stroke and potential mechanisms from the perspective of the gut-brain axis, providing a solid scientific basis for its clinical use.

## Materials and methods

### Drugs and reagents

Nimodipine Tablets (20 mg/tablet, H44025019) were purchased from Huanan Pharmaceutical (Dongguan, China). Indole lactic acid (M66846) was purchased from Meryer Biochemical Technology (Shanghai, China). 2, 3, 5-triphenyltetrazolium chloride was purchased from Sigma-Aldrich (USA). Occludin (Cat# DF7504, RRID: AB_2841004.), Claudin-1 (Cat# AF0127, RRID: AB_2833311.) and ZO-1 (Cat# AF5145, RRID: AB_2837631.) were purchased from Affinity Biosciences (USA). S-100B (MM-20763R1), NGB (MM-0332R1), DAO (MM-21169R1), LPS (MM-0647R1), D-lactate (MM-21239R1) and ILA (MM-72080R1) ELISA kits were purchased from Jiangsu Meimian Industrial Co., Ltd. (Jiangsu, China). SuperMix for qPCR (R223-01) and Master Mix (Q311-02) were purchased from Vazyme (Nanjing, China). Brain Heart Infusion Broth (028360) was purchased from HuanKai Microbial (Guangzhou, China).

### Preparation of BHTD

BHTD consists of the following dried herbal components: 15 g of Astragali Radix, 10 g of Chuanxiong Rhizoma, 10 g of Salviae Miltiorrhizae Radix et Rhizoma, 9 g of Notoginseng Radix et Rhizoma, 12 g of Paeoniae Radix Rubra, 10 g of Pheretima, 15 g of Poria, 15 g of Puerariae Lobatae Radix, 9 g of Pinelliae Rhizoma Praeparatum, and 10 g of Glycyrrhizae Radix et Rhizoma Praeparata Cum Melle (Details for Table S1). We have verified the names of the plants on http://mpns.kew.org. BHTD was obtained from the Integrated Traditional Chinese and Western Medicine Hospital of Southern Medical University. After immersing in distilled water for one hour, the herbs were twice decocted for one hour (1:10, w/v). The filtrates were concentrated at 0.259, 0.5175 and 1.035 g/mL, respectively. The dosage of BHTD used in rats was converted from the clinical dosage using the following formula: The dosage of BHTD-H = 6.3 × 115 g/ 70 kg [33]. The equivalent dose ratio for humans and rats was 6.3. The dosage of clinical raw drug was 115 g/ person/ day. The average body weight of normal adults was 70 kg.

The detection of BHTD was performed using LC–MS/MS (Orbitrap Exploris 240; Thermo Fisher, USA). The LC gradients and MS conditions were shown in Table S2. The result of LC–MS/MS was shown in Fig. S1.

### Animals and establishment of model

Male Sprague–Dawley rats (200 ± 20 g) were supplied by the central animal facility of Southern Medical University (License Number: SCXK (Guangdong) 2021-0041) and housed at Southern Medical University Experimental Animal Center (Animal Utilization Permit Number: SYXK (Guangdong) 2021-0167). They had free access to food and water with a 12 h light/dark cycle and a constant temperature (23 °C).

The cerebral ischemia model of rats was established by the middle cerebral artery occlusion (MCAO) method [34]. The rats were anesthetized by inhalation of isoflurane. A monofilament was inserted from the external carotid artery, through the internal carotid artery, and into the middle cerebral artery. To create reperfusion, the monofilament was removed after two hours of occlusion. The sham group underwent the identical processes, except that monofilament was not inserted.

### Experiment design

Rats were divided into 6 experimental groups: sham-operated group (Sham, n = 10), model group (MCAO, n = 25), 2.59 g/kg BHTD treatment group (BHTD-L, n = 20), 5.175 g/kg BHTD treatment group (BHTD-M, n = 18), 10.35 g/kg BHTD treatment group (BHTD-H, n = 13) and 10 mg/kg Nimodipine treatment group (Nimodipine, n = 14). The BHTD treatment group and the Nimodipine treatment group were given oral doses once a day for 14 days. The sham-operated and the model group were gavaged with an equivalent volume of distilled water.

Fecal Microbiota Transplantation (FMT) was based on previous studies [29]. Fecal material from MCAO and BHTD-H groups was resuspended in PBS (100 mg/mL). A new batch of the model rats was separated into two groups: MCAO-recipient group (FMT-MCAO) and BHTD-recipient group (FMT-BHTD). Two groups were orally administered antibiotics once a day to clear the gut microbiota during the first 4 days and 2 mL of fecal material once a day for 10 days.

In the ILA group, rats after MCAO operation were administered 13.84 mg/kg of indole lactic acid once a day for 14 days [35].

### Evaluation of neurological defects

Neurological defects were assessed by Longa and modified neurologic severity score (mNSS) [34, 36, 37]. The Longa score is a classic neurological assessment method. Besides, mNSS was applied to evaluate a combination of movement, sensation, balance and reflexes before MCAO surgery and on days 1, 3, 7, and 14 of treatment. The specific scoring rules are shown in Table S3.

### Measurement of infarct volume

The infarct volume was measured by TTC staining. The entire brain was cut coronally into 2 mm slices, then immersed in 2% TTC at 37 °C for 15 min. The area of infarction was analyzed with Image J analysis software (version 6.0, NIH). The cerebral infarction ratio is calculated as follows: infarct size/the size of the non-ischemic hemisphere × 100% [38].

### Haematoxylin–eosin (HE), Nissl and Immunohistochemical staining

For HE and Nissl staining, fresh brain and colon tissues were promptly fixed in 4% paraformaldehyde solution, then dried in a graded ethanol series (70–100%) and embedded in paraffin. The embedded samples were stained with HE and Nissl staining solutions, respectively.

For immunohistochemistry, brain and colon tissue samples were incubated with different antibodies. The results were analyzed by Image-Pro Plus 6.0 software (Media Cybernetics Inc., USA).

### Biochemical analysis

The measurement of the serum levels of T-CHO, TG, LDL and HDL was quantified according to the kit protocols (Nanjing Institute of Bioengineering, China).

### Enzyme-linked immunosorbent assay (ELISA)

The serum levels of S-100B, NGB, DAO, LPS, and D-lactate and the brain levels of S-100B, NGB, and ILA were measured according to the ELISA kits, respectively.

### Intestinal permeability test

Rats were administered FD-4 orally (200 mg/kg). After 4 h, collected plasma was used to measure the level of FD-4 by a multimode microplate reader (Tecan, Switzerland) (excitation wavelength: 485 nm, emission wavelength: 530 nm). [39]

### Quantitative real-time PCR (qRT–PCR) assay

Using the Animal Total RNA Isolation Kit, total RNA was extracted. Reverse-transcription reactions were carried out to produce cDNA using SuperMix for qPCR. qRT-PCR measurements were carried out on the LightCycler480 using Master Mix. β-actin was used as a control gene. The data were calculated by the comparative 2 − ∆∆CT method. The primer sequences are shown in Supplementary Table S4.

### Gut microbiota analysis

16S rRNA gene sequencing was conducted by Shanghai Majorbio Bio-pharm Technology Co., Ltd. The V3-V4 variable region was PCR amplificated. The forward primer was 338F (5ʹ—ACTCCTACGGGAGGCAGCAG—3ʹ) and the reverse primer was 806R (5ʹ—GGACTACHVGGGTWTCTAAT—3ʹ). The sequence data were analyzed using Illumina's PE300/PE250 platforms. OTU clustering is based on 97% similarity. Alpha diversity index was computed by mothur software (version 1.30.2); similarity of microbial structure based on binary_jaccard distance algorithm; LEfSe analysis showed differences in genus-level abundance (LDA > 2, p < 0.05); spearman's correlation based on |r|> 0.6 and p < 0.05; redundancy analysis (RDA) investigated the indicators on the structure of the gut microbiota; predictive analysis of 16S function was performed using PICRUSt2 software (version 2.2.0).

### Metabolomics analysis

The metabolomics procedure was performed by Metabo-Profile Biotechnology Co. Ltd. (Shanghai, China). When sacrificing at the end of animal experiments, collect fresh fecal samples into tubes and quickly place them in liquid nitrogen to reduce degradation. Weigh 50 mg of fecal sample into a centrifuge tube, add 400 µL of extraction solution (methanol:acetonitrile = 1:1 (v:v)) containing 0.02 mg/mL of internal standard (L-2-chlorophenylalanine), homogenize for 3 min, shake for 15 min, place the sample at − 20 °C for 20 min, and then centrifuge the sample for 20 min at 4 °C for 18,000 g. Pipette the supernatant into the injection bottle and wait for the sample to be analyzed. The supernatant was pipetted into a vial to be analyzed. Mobile phase A was 95% water plus 5% acetonitrile with 0.1% formic acid. Mobile phase B was 47.5% acetonitrile + 47.5% isopropanol + 5% water with 0.1% formic acid. The gradient elution is based on a previous study [40]. Statistical analysis and pathway analysis were handled using MetaboAnalyst 5.0.

Indole lactic acid was detected by UPLC-MS/MS. Mobile phase A was water. Mobile phase B was 70% acetonitrile + 30% IPA. The gradient elution process is based on a previous study [41]. Data were processed by MassLynx software (v4.1, Waters, Milford, MA, USA).

### In vitro* fermentation experiment*

The in vitro fermentation experiment was conducted in accordance with the earlier description [42]. Fresh cecum contents from stroke rats were resuspended in BHI Medium (50 mg/mL) and co-cultured with or without BHTD under anaerobic conditions (80% N_2_, 10% H_2_, 10% CO_2_). The dose of BHTD was based on previous studies [43]. Samples were obtained at 0, 24 and 48 h of incubation, centrifuged at 13,000 rpm for 5 min at 4 °C, and the supernatant was used for determination.

### Statistical analysis

The data were analyzed by the IBM SPSS 25.0 statistical software and plotted by GraphPad Prism version 8.0. Data are expressed as mean ± standard deviation (SD). Neurological score was evaluated using Kruskal–Wallis test. Significant differences between the two groups were evaluated by a two-tailed unpaired Student’s t test, and in more than two groups were evaluated by one-way or two-way analysis of variance (ANOVA) followed by Tukey’s multiple comparisons test. Significant differences were considered at *P < 0.05, **P < 0.01 and ***P < 0.001. The variable importance projection (VIP) produced by OPLS-DA > 1 was considered statistically significant.

## Results

### BHTD ameliorated neurological function, reduced infarct volume and improved dyslipidemia

**Fig. 1:**
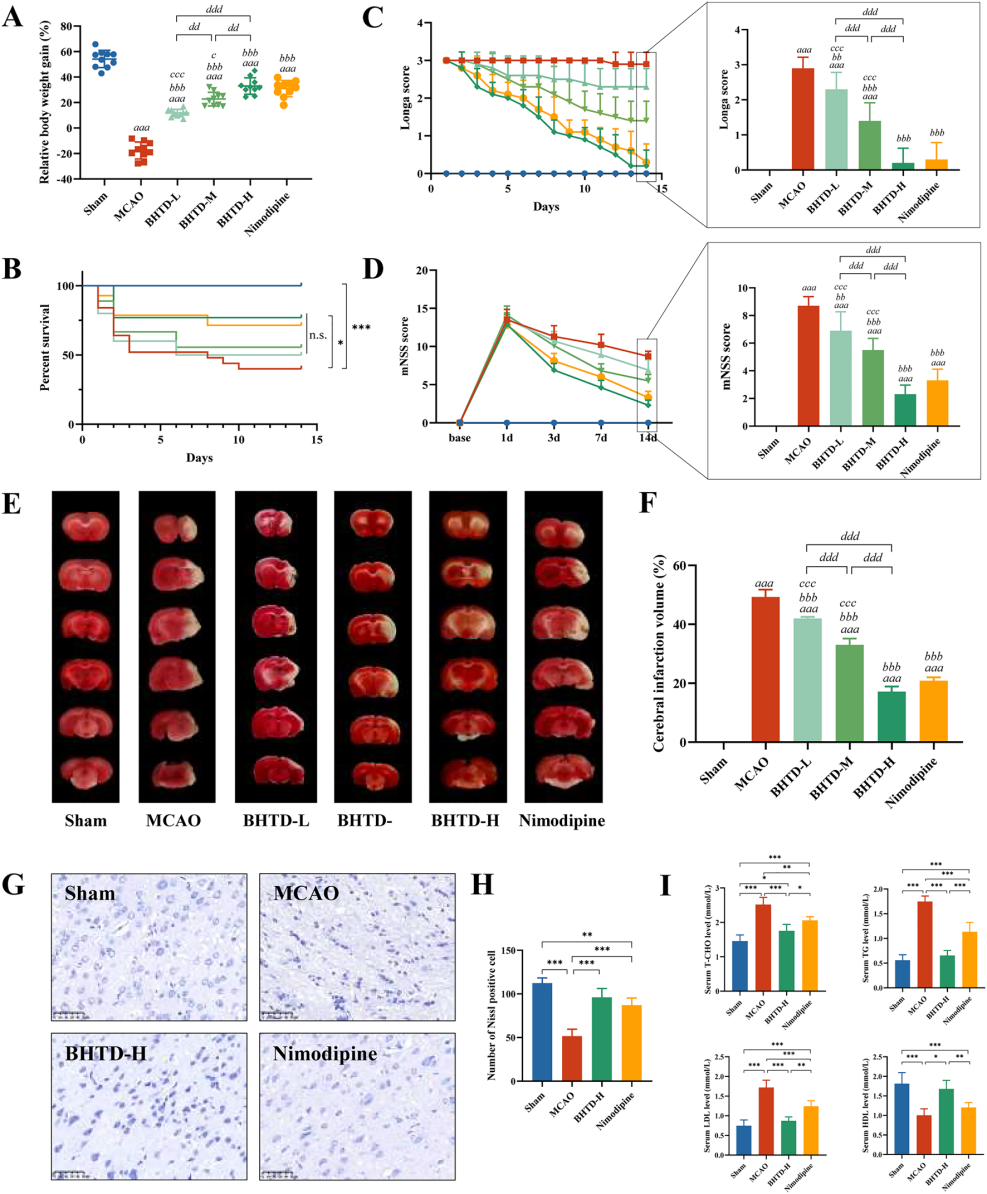
BHTD ameliorated neurological function, reduced infarct volume and improved dyslipidemia. **A** Relative body weight gain for 14 days. **B** Survival curve. **C** Longa score. **D** mNSS score. (**A**–**D**, n = 10) **E** Representative images of TTC staining. **F** Quantitative analysis of cerebral infarct areas (n = 4). **G** Representative images of Nissl staining (scale bar = 50 µm). **H** Quantification of the Nissl staining (n = 4). **I** Blood lipid in serum: T-CHO, TG, LDL and HDL (n = 6). All data are shown as mean ± SD. For **A**–**D**, ^a^P < 0.05, ^aa^P < 0.01 and ^aaa^P < 0.001, vs the Sham group; ^b^P < 0.05, ^bb^P < 0.01 and ^bbb^P < 0.001, vs the MCAO group; ^c^P < 0.05, ^cc^P < 0.01 and ^ccc^P < 0.001, vs the Nimodipine group; ^d^P < 0.05, ^dd^P < 0.01 and ^ddd^P < 0.001, compared in three BHTD groups. For **E**–**I**, ^*^P < 0.05, ^**^P < 0.01, ^***^P < 0.001

We first investigated the effects of low, medium and high doses of BHTD on ischemic stroke in rats. The MCAO group showed a significant reduction in body weight gain at 14 days post-surgery, whereas body weight loss due to stroke was mitigated by the administration of various doses of BHTD or Nimodipine, and weight gain in the BHTD-H group was higher than in all other intervention groups (Fig. 1A). In addition, a noteworthy rise in survival rate was noted between the MCAO and BHTD-H groups (Fig. 1B). The Longa score and the mNSS score were used to assess the impact of recovery on neurological function. During the observation period, the Longa score of the MCAO group was maintained at about 3 points and the mNSS score was maintained above 8 points, indicating that the neural dysfunction could not recover spontaneously (Fig. 1C, D). However, the neurologic impairment recovered over time after the intervention of different doses of BHTD and Nimodipine (Fig. 1C, D). On the last day of the experimental observation period, neurologic function scores were lowest in the BHTD-H group, which did not differ substantially from the Nimodipine group, suggesting that the high dose of BHTD achieved the best recovery of neurologic function among the three groups (Fig. 1C, D).

According to the result of TTC staining, all doses of BHTD or Nimodipine reduced the areas of white infarcts and collapse (Fig. 1E). Among them, the BHTD-H group reduced the infarction rate to 17.20% and the area of cerebral infarction was smaller than all other intervention groups (Fig. 1F). Based on the above, we determined that the high dose of BHTD exhibited the best protective brain injury efficacy of the three and evaluated it in subsequent experiments. Nissl staining exhibited that the number of Nissl-positive cells in the ischemic penumbra of the MCAO group significantly decreased, whereas this was restored by the intervention of BHTD and Nimodipine (Fig. 1G–H). This suggested that BHTD could significantly improve the reduction in the number of neuronal cells in the ischemic penumbra (Fig. 1H). Dyslipidemia is an independent risk factor of ischemic stroke, typically linked to thrombosis [44]. Serum T-CHO, TG and LDL levels were significantly higher while HDL levels were lower in the MCAO group than the Sham group, indicating that ischemic stroke led to abnormal lipid metabolism, which increased the risk of thrombosis and thus slowed the progression of the disease. However, such dyslipidemia was improved after BHTD or Nimodipine supplementation and BHTD showed better lipid regulation than Nimodipine (Fig. 1I). Collectively, these results suggested that BHTD efficiently increased the number of neurons to relieve neurological function, reduced the volume of cerebral infarction, and alleviated ischemic stroke-induced dyslipidemia.

### BHTD reduced cerebral tissue damage and repaired the blood–brain barrier

**Fig. 2:**
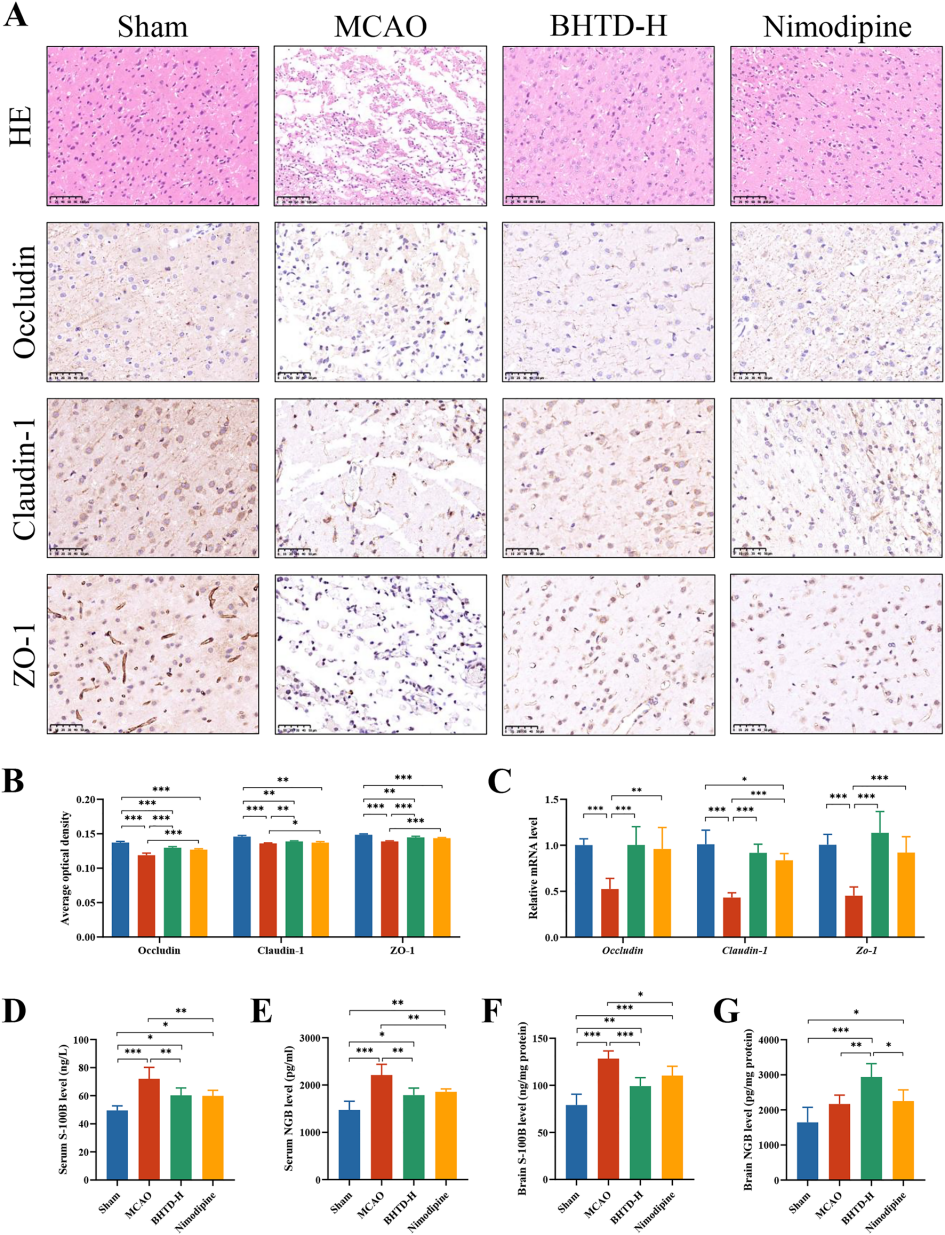
BHTD alleviated brain injury and repaired the blood–brain barrier. **A** Representative images of HE staining and IHC staining in brain (× 200, scale bar = 100 µm). **B** The average optical densities of Occludin, Claudin-1 and ZO-1 in brain. (n = 4) **C** Relative mRNA level of *Occludin*, *Claudin-1* and *Zo-1* in brain. (n = 6) **D**, **E** Serum levels of S-100B and NGB. **F**, **G** Brain levels of S-100B and NGB. **D**–**G**, n = 6) All data are shown as mean ± SD. ^*^P < 0.05, ^**^P < 0.01, ^***^P < 0.001

HE staining showed the cerebral cortex cells were neatly arranged with normal structures in the Sham group, while brain tissue liquefaction, cortical disorganization and cell necrosis were observed in the MCAO group. BHTD significantly ameliorated such pathological injury of brain tissue, as evidenced by the rearranged nerve cells and clear cell outline (Fig. 2A). Brain tissue injury means the collapse of the blood–brain barrier (BBB), which is a pathogenic feature during an ischemic stroke [45]. The tight junction between the cells ensures the BBB's physical barrier function [46]. Therefore, we assessed the concentrations of three tight junction proteins (TJPs), Occludin, Claudin-1 and ZO-1. Immunohistochemical (IHC) analysis illustrated that the levels of Occludin, Claudin-1 and ZO-1 in the MCAO group were significantly decreased (Fig. 2A, B). However, BHTD treatment markedly reversed such loss (Fig. 2A, B). As predicted, mRNA expression matched the IHC results (Fig. 2C). These results indicated that BHTD intervention could repair brain tissue damage and promote the structural integrity of the BBB.

BBB could restrict the transportation of peripherally hazardous substances, thus maintaining the stability of the central internal environment [45]. Thus, the indicators of brain tissue damage were estimated to assess the function of the BBB. S-100B is a brain damage marker that is significantly increased in areas of infarcted tissue, and neuroglobin (NGB) is a neuroprotective protein expressed primarily in neurons [47, 48]. As centrally specific substances and macromolecular proteins, both of them would be released from brain into the bloodstream through the breached blood–brain barrier under conditions of ischemic stroke [3, 49]. According to the ELISA results, the MCAO group had significantly higher serum levels of S100B and NGB, and these levels significantly decreased following the administration of BHTD (Fig. 2D, E). These data indicated that BHTD could repair the disrupted function and increased permeability of the BBB induced by ischemic stroke. Moreover, the levels of S100B and NGB in the brain were measured. The levels of S-100B in brain were significantly increased in the MCAO group. After BHTD administration, its levels were markedly reduced (Fig. 2F). The brain levels of NGB in the MCAO group was somewhat greater than in the Sham group because of the reflexive increase in acute ischemia and hypoxia [48]. The BHTD-H group exhibited a considerably greater level compared to the Sham and MCAO groups (Fig. 2G). These results suggested that BHTD could reduce S100B in the infarcted area and increase the content of NGB in brain, providing a shielding influence on brain tissue and neurons. Altogether, BHTD could repair pathologic brain tissue damage, increase expression of tight junction proteins, and preserve the BBB’s structural and functional integrity.

### BHTD repaired the intestinal barrier and reduced intestinal bacterial translocation

**Fig.3:**
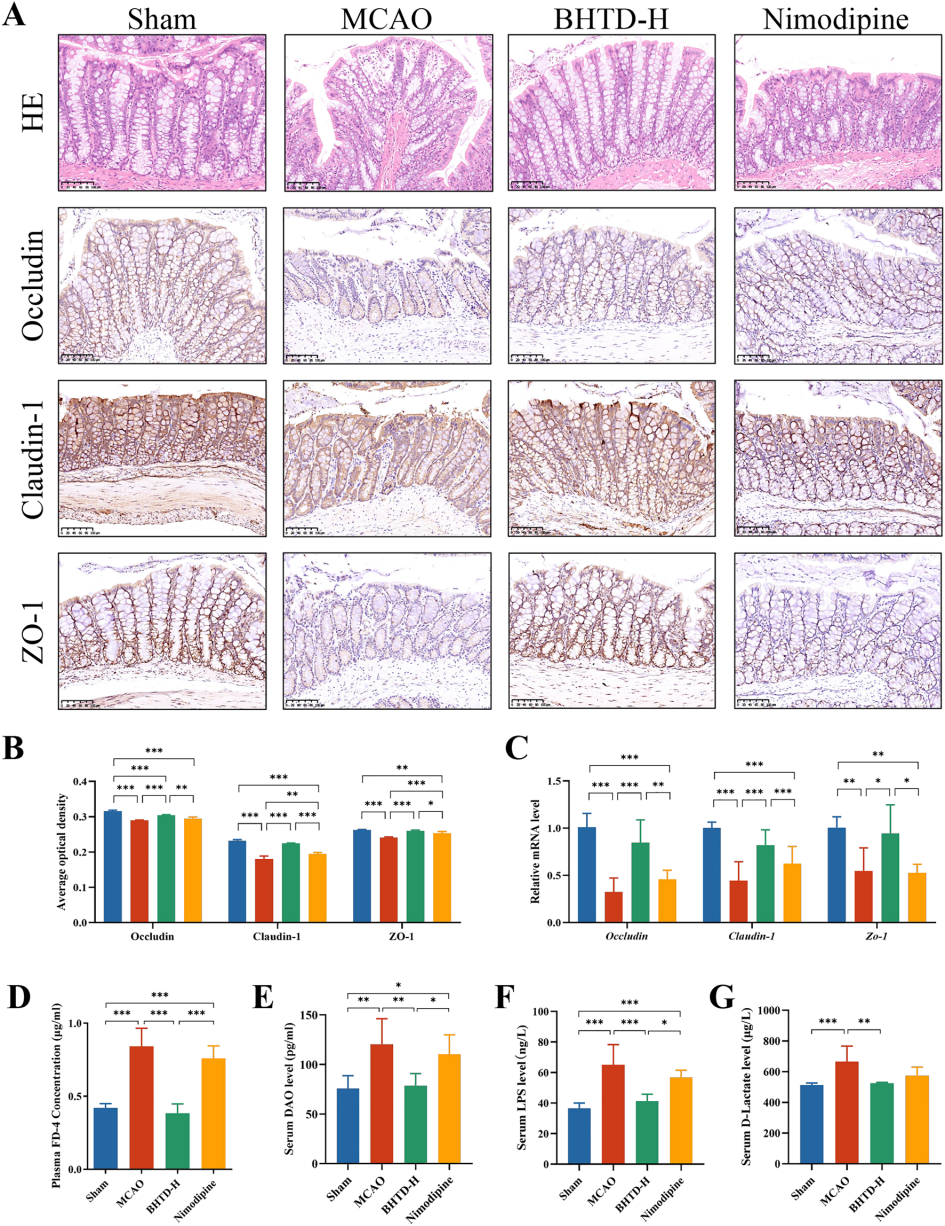
BHTD reduced intestinal bacterial translocation and intestinal permeability, and repaired colonic damage. **A** Representative images of HE and IHC staining in colon (scale bar = 100 µm). **B** The average optical densities of Occludin, Claudin-1 and ZO-1 in colon (n = 4). **C** Relative mRNA levels of *Occludin*, *Claudin-1* and *Zo-1* in colon (n = 6). **D** Plasma levels of FD-4. **E**, **F**, **G** The serum levels of DAO, LPS, D-lactate. (**D**–**G**, n = 6) All data are shown as mean ± SD. ^*^P < 0.05, ^**^P < 0.01, ^***^P < 0.001

After cerebral ischemia, there is often a stress response in the gut that leads to dysregulation of gut homeostasis, which can further worsen ischemic stroke and lengthen the disease's course [23, 50]. HE staining of the colon demonstrated thinning of the mucosal layer and necrosis of epithelial cells at 14th day after MCAO (Fig. 3A). Moreover, there is a loss of goblet cells in the Nimodipine group (Fig. 3A). This finding showed that ischemic stroke could induce colonic injury in rats, which was unaffected by Nimodipine. However, BHTD supplementation increased tight epithelial cell arrangement and decreased goblet cell loss (Fig. 3A). In addition, we also assessed the levels of TJPs in the colon. Consistent with the brain, both IHC results and qPCR results showed that the Occludin, Claudin-1 and ZO-1 levels were significantly lower in the MCAO group; however, these levels were greatly restored by BHTD (Fig. 3A–C). Notably, alterations in these tight junction protein levels between the gut and brain were found to significantly positively correlate (Fig. S2). The results indicated that the blood–brain barrier was closely associated with the intestinal barrier while BHTD intervention was effective in improving both. Colonic injury is characterized by disrupted gut barrier and increased intestinal permeability, which allow the gut microbiota or its products to translocate into extraintestinal tissues [51]. Thus, the markers of gut permeability and bacterial translocation were evaluated. The MCAO group had considerably greater plasma FD-4 and serum levels of DAO, LPS, and D-lactate than the Sham group; however, the levels of these indicators were considerably lowered by the BHTD intervention (Fig. 3D–G). These results suggested that BHTD could alleviate ischemic stroke-induced colon damage and repair the intestinal barrier, thereby inhibiting intestinal bacterial translocation.

### BHTD reversed ischemic stroke-induced gut microbial dysbiosis

**Fig. 4:**
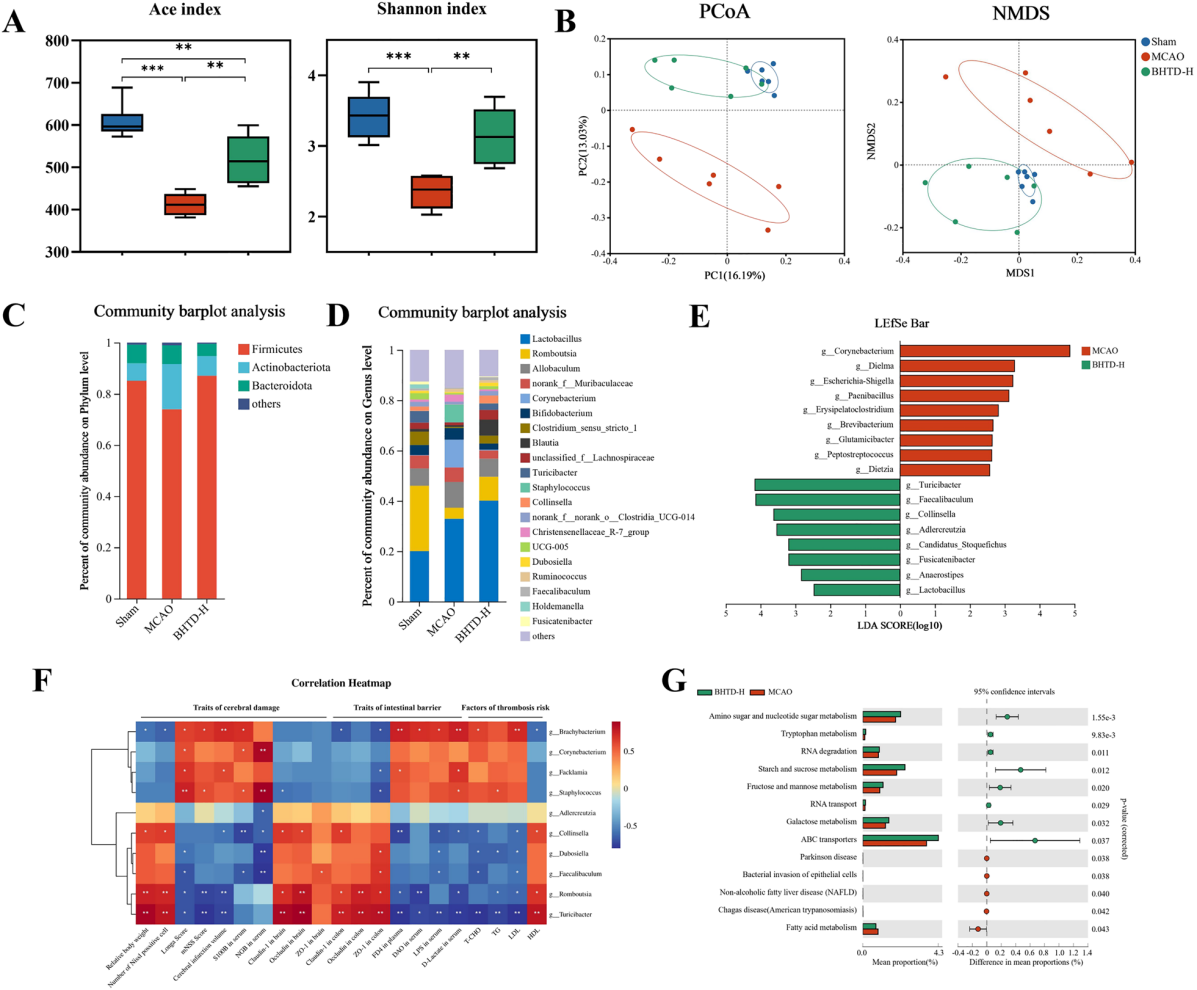
BHTD reversed ischemic stroke-induced gut microbial dysbiosis. **A** α-Diversity (n = 6). **B** β-Diversity (n = 6). **C** Relative abundance at the Phylum level. **D** Relative abundance at the Genus level. **E** LEfSe analysis. **F** Correlation heatmap. **G** KEGG functional pathway based on PICRUSt2. Data are expressed as the mean ± SD. ^*^P < 0.05, ^**^P < 0.01, ^***^P < 0.001

Gut microbiota is an integral part of the intestinal barrier [52]. Therefore, in order to evaluate the composition and function of the gut microbiota, we employed 16S rRNA gene sequencing. The gut microbiota's richness and diversity were severely reduced in the MCAO group, as indicated by the ACE and Shannon indices, and they significantly improved following BHTD treatment (Fig. 4A); observed species, Chao1 index and Simpson index exhibited the same results (Fig. S3A–C). Then, the β diversity was examined with PCoA and NMDS (Fig. 4B). The results showed that the Sham and the MCAO groups were clearly separated, exhibiting that ischemic stroke led to disorganization and structural changes of the gut microbiota. After BHTD treatment, the gut microbial composition of rats significantly changed and converged to that of the Sham group, indicating that BHTD could remodel the gut microbial structure to normal levels.

Additionally, a comparison was made between the relative abundances of the phylum and genus. The MCAO group rats had fewer *Firmicutes* and more *Actinobacteria*; this difference was corrected by BHTD (Fig. 4C). The genus level reflected that the MCAO group had larger abundances of harmful bacteria like *Corynebacterium* and *Staphylococcus*, while the BHTD group had an enrichment of beneficial bacteria including *Romboutsia*, *Turicibacter* and *Collinsella* (Fig. 4D). LEfSe analysis was applied to identify the biomarkers separating the MCAO group from the BHTD-H group (Fig. 4E and Fig. S3D). Pathogens or opportunistic pathogens, including *Corynebacterium*, *Dielma*, *Escherichia-Shigella*, *Paenibacillus*, *Peptococcus* and *Quinella*, were enriched in the MCAO group; however, BHTD intervention resulted in an enrichment of beneficial bacteria *Turicibacter*, *Collinsella* and SCFA-producing bacteria, such as *Faecalibaculum*, *Adlercreutzia*, *Fusicatenibacter* and *Lactobacillus* (Fig. 4E). Next, Spearman’s correlation was computed to evaluate the relationship between specific gut microbiome and efficacious parameters, including traits of cerebral damage and intestinal barrier and risk factors for thrombosis (Fig. 4F). Moreover, the Redundancy Analysis (RDA) further revealed the relationship between the gut microbiota and the efficacy parameters among the Sham, MCAO and BHTD-H groups, respectively (Fig. S3F). The findings showed that the indicators linked to the amelioration of ischemic stroke were inversely correlated with the quantity of harmful bacteria and positively correlated with the quantity of beneficial bacteria (Fig. 4F and Fig. S3F). These results suggested that BHTD could remodel the gut microbiota, reverse microbiota disruption, and enrich beneficial bacteria, thereby promoting the recovery of ischemic stroke.

The remodeled gut microbiota represents a novel physiological function [53]. Hence, PICRUSt2 analysis and KEGG pathway analysis were performed to elucidate how BHTD affects microbial physiological activity. 13 significantly different pathways were identified between the MCAO and BHTD-H groups (Fig. 4G). Notably, the MCAO group exhibited a notable increase in “Bacterial invasion of epithelial cells,” representing that bacteria that might be damaging to intestinal epithelial cells increased after ischemic stroke. Furthermore, we found that BHTD bolstered the microbial functions linked to the metabolism of amino acids, such as “Amino sugar and nucleotide sugar metabolism” and “Tryptophan metabolism”; “Starch and sucrose metabolism” and “Fructose and mannose metabolism”, associating with the metabolism of nutrients, which were markedly enriched in the BHTD-H group (Fig. 4G). This result suggested BHTD supplement enhanced microbial functions in amino acid metabolism and nutrient metabolism. On the whole, BHTD boosted the quantity of beneficial bacteria to reverse the dysbiosis of the gut microbiota brought on by ischemic stroke, and gut microbiota remodeling along with new physiological functions might be a target for BHTD to alleviate ischemic stroke.

### Transplantation of BHTD-regulated gut microbiota relieved the symptoms of ischemic stroke

**Fig. 5:**
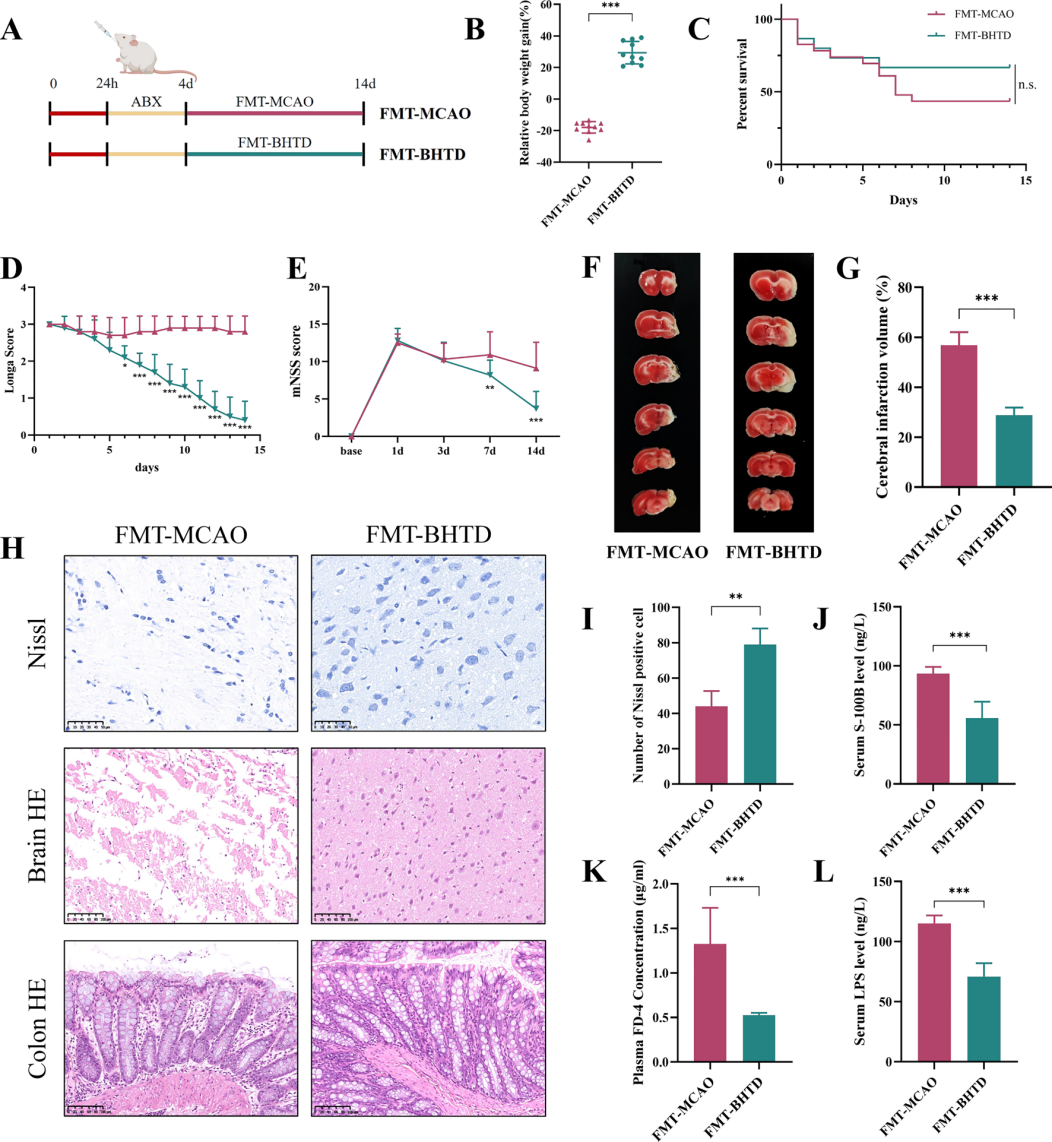
Transplantation of BHTD-regulated gut microbiota relieved the symptoms of ischemic stroke. **A** The schematic diagram of the experimental procedure. **B** Relative body weight gain for 14 days. **C** Survival curve. **D** Longa score. **E** mNSS score. (**A**–**D**, n = 10) (**F**) Representative images of TTC staining. (G) Quantitative analysis of cerebral infarct areas (n = 4). **H** Representative images of Nissl (scale bar = 50 µm), HE (scale bar = 100 µm) staining in brain and colon tissues. **I** Quantification of the Nissl staining (n = 4). **J** Serum levels of S-100B. **K** Plasma levels of FD-4. **L** Serum levels of LPS. **J**–**L**, n = 6) All data are shown as mean ± SD. ^*^P < 0.05, ^**^P < 0.01, ^***^P < 0.001.

FMT experiment was conducted in order to discover further about the impact of the gut microbiota modulated by BHTD. After receiving a combination of antibiotics orally for four days to deplete the gut microbiota, rats were separately administered fecal suspensions from MCAO and BHTD-H group rats (Fig. 5A). On the 14th day, the FMT-BHTD group rats’ body weight gain was considerably higher than the FMT-MCAO group (Fig. 5B). Despite the fact that there was no apparent difference in the survival curves between the two groups, FMT-BHTD had a higher survival rate than FMT-MCAO (Fig. 5C). In addition, both the Longa score and the mNSS score indicated that transplantation of BHTD-regulated microbiota significantly reduced neurological function scores. Interestingly, no discernible difference was observed between the FMT-MCAO and FMT-BHTD groups within the first 4 days in survival rate, Longa score or mNSS score. Nevertheless, after transplantation of microbiota from different groups, a decreased survival rate and increased neurological function scores were noticed in the FMT-MCAO group, while the indicators of the FMT-BHTD group recovered (Fig. 5C–E). TTC staining revealed that tissue collapse and cerebral infarction due to stroke could be alleviated by BHTD-regulated microbiota (Fig. 5F, G). Nissl and HE staining of the brain tissue demonstrated that more Nissl-positive cells and less necrosis and edema in the brain tissue were observed in the FMT-BHTD group (Fig. 5H, I). Furthermore, HE staining of colon tissue showed that increased goblet cells and a thickening mucosal layer were noticed (Fig. 5H). Likewise, the results of reduced serum S100B, plasma FD-4, and serum LPS levels showed transplantation of BHTD-regulated microbiota markedly repaired the blood–brain barrier, reduced intestinal permeability and decreased intestinal bacterial translocation (Fig. 5J–L). These results suggested that transplanting the fecal microbiota of rats in the BHTD-H group achieved comparable protective effects as supplementation with BHTD, indicating that remodeling the gut microbiota was an upstream factor for BHTD against ischemic stroke.

### BHTD upregulated tryptophan metabolism in the gut microbiota to promote the production of indole lactic acid

**Fig. 6:**
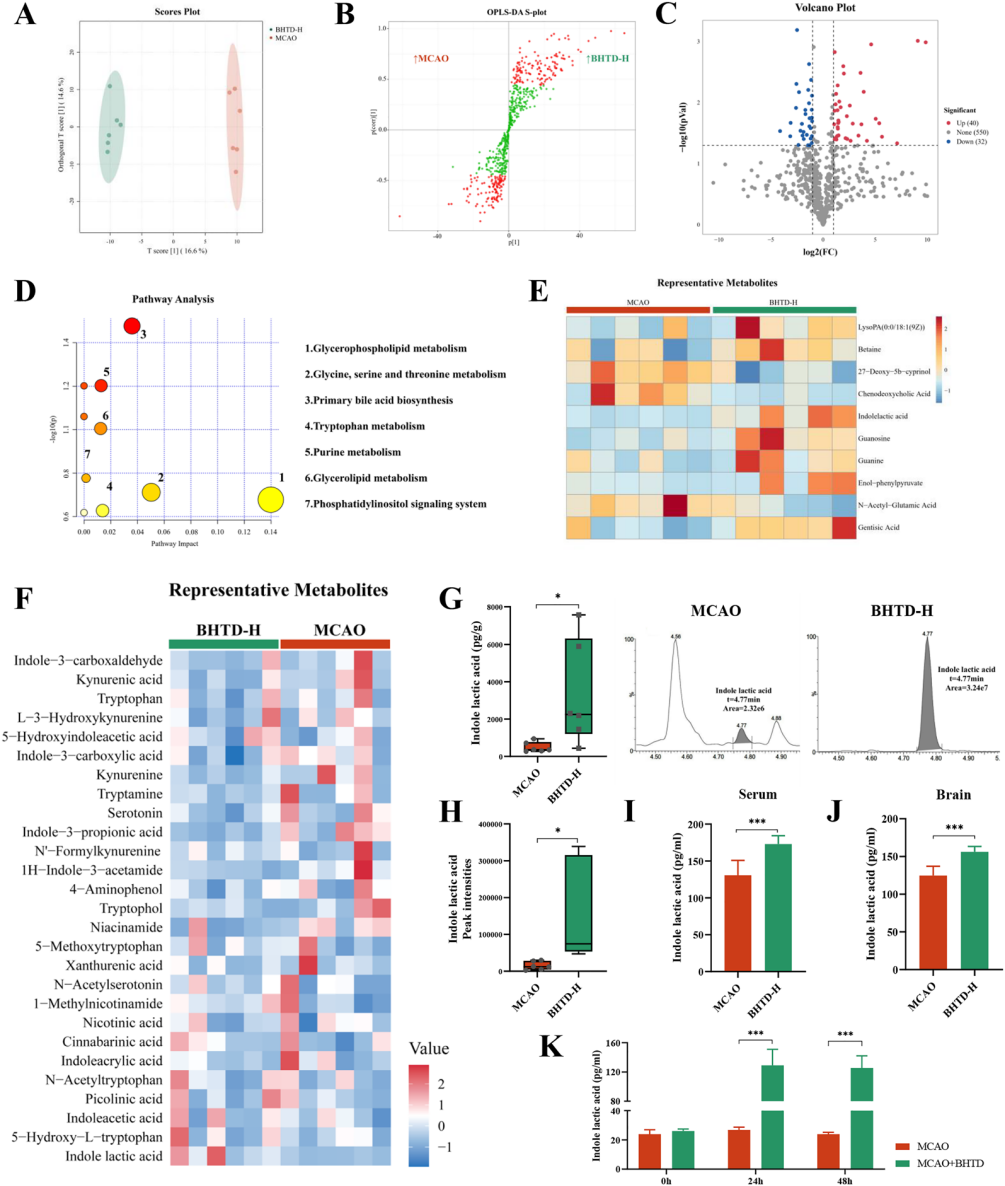
BHTD upregulated tryptophan metabolism in the gut microbiota to promote the production of indole lactic acid. **A** OPLS-DA plot (n = 6). **B** S-plot analysis. **C** Volcano plot. **D** Pathway analysis based on KEGG database. **E**–**F** Heatmap of metabolites. **G** The levels of ILA in the cecal contents (n = 6) and representative chromatogram. **H** The peak intensities of ILA (n = 6). **I**, **J** The levels of ILA in the serum and brain (n = 6). **K** The levels of ILA in the in vitro fermentation assay (n = 6). All data are shown as mean ± SD. ^*^P < 0.05, ^**^P < 0.01, ^***^P < 0.001

Non-targeted metabolomic study was performed to investigate the mechanism of BHTD in the gut microbiota. The result of OPLS-DA revealed metabolite profiles that were substantially different between the MCAO and BHTD-H groups, suggesting that BHTD had changed the metabolic profiles (Fig. 6A). The OPLS-DA model had a goodness-of-fit value (R^2^Y) of 0.983 (very close to 1) and a goodness-of-prediction value (Q^2^Y) of 0.583 (> 0.4), indicating it had high reliability (Fig. S4A). Based on the variable importance in the projection (VIP) values calculated from the OPLS-DA model, the s-plot presented 125 differentially up-regulated and 131 differentially down-regulated metabolites between the MCAO and BHTD-H groups (Fig. 6B). Moreover, as demonstrated by the volcano plot, setting the screening criteria as fold change ≥ 2 and p ≤ 0.05, 72 differential metabolites (32 down-regulated and 40 up-regulated) were screened between the MCAO group and the BHTD-H group (Fig. 6C). Following the screening of all differential metabolites, the KEGG database was used to conduct pathway enrichment analysis. 10 KEGG pathways were identified and we labeled 7 main pathways (Fig. 6D). Heatmap analysis revealed 10 representative metabolites matching the KEGG pathway (Fig. 6E). The “tryptophan metabolism” pathway was altered (Fig. 6D), which matches the results of microbial PICRUSt2 analysis (Fig. 4G). Tryptophan metabolism is involved in regulating mood and promoting brain development, which is closely associated with central nervous system disorders [54]. Moreover, a tryptophan metabolite, melatonin, has been proven to be effective in relieving ischemic stroke in our previous study [29]. Therefore, we focused on tryptophan metabolism. We further quantified the levels of 27 tryptophan metabolites in the cecal content by targeted metabolomic analysis (Fig. 6F). The results showed that 3 indole metabolites were significantly changed after BHTD intervention and only indole lactic acid (ILA) was upgraded (Fig. 6G and Fig. S4B), which were consistent with non-targeted metabolomic analysis (Fig. 6H). Furthermore, levels of ILA in the serum and brain were detected and were significantly higher in the BHTD-H group (Fig. 6I, J). Next, to explore the source of ILA, we carried out an in vitro fermentation experiment (Fig. S3C). More ILA was formed by the gut microbiota co-cultivating with BHTD than that cultured alone in the medium, indicating that the gut microbiota can synthesize ILA and that BHTD could drive the gut microbiota to produce more ILA directly (Fig. 6K). Altogether, BHTD could upgrade tryptophan metabolism in the gut microbiota to increase the level of ILA in the gut, serum and brain, which might be the key to the efficacy of BHTD by modulating the gut microbiota.

### Gavage of indole lactic acid protected against ischemic stroke

**Fig.7:**
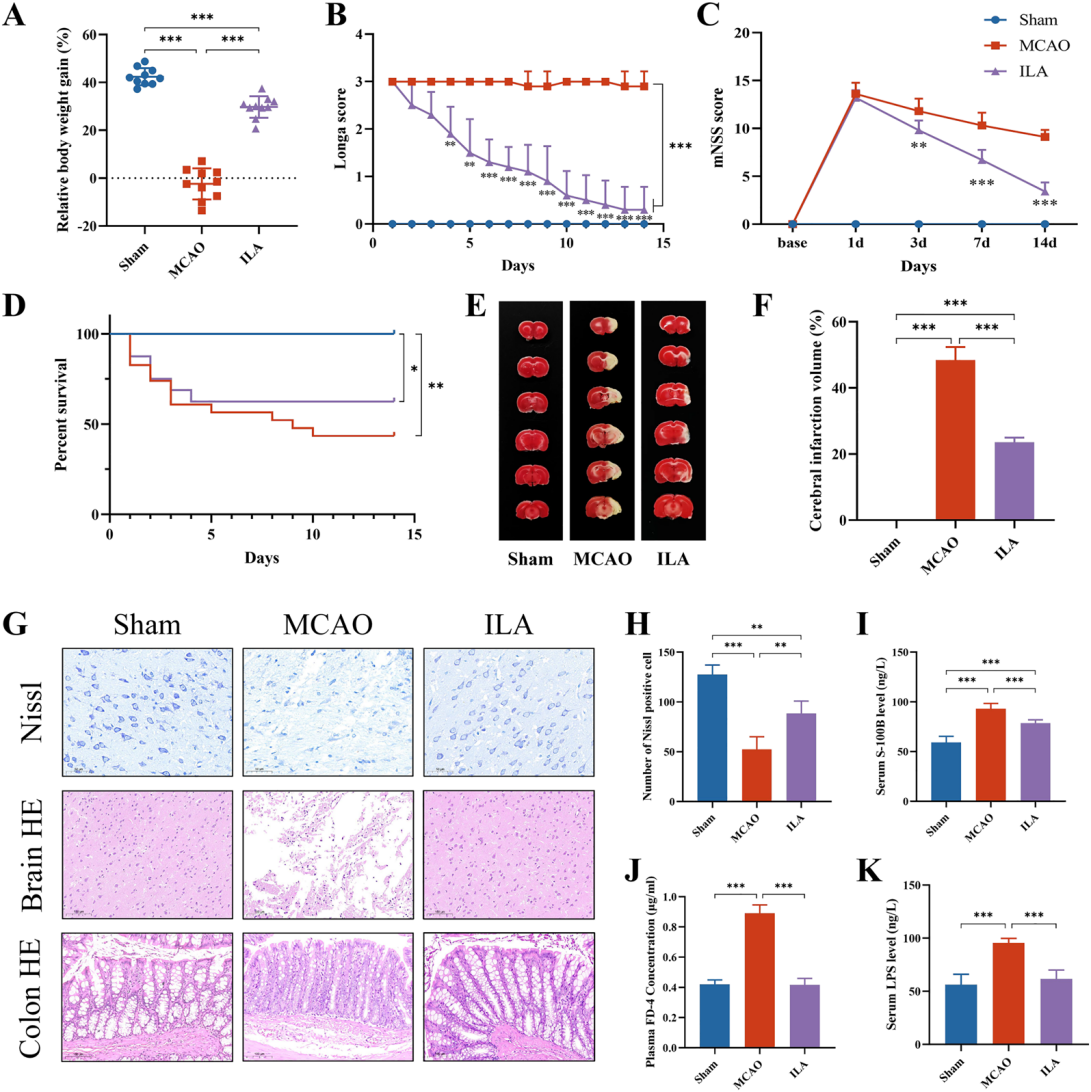
Gavage of indole lactic acid protected against ischemic stroke. **A** Relative body weight gain for 14 days. **B** Longa score. **C** mNSS score. **D** Survival curve. (A-D, n = 10) **E** Representative images of TTC staining. **F** Quantitative analysis of cerebral infarct areas (n = 4). **G** Representative images of Nissl (scale bar = 50 µm), HE (scale bar = 100 µm) staining in brain and colon tissues. **H** Quantification of the Nissl staining (n = 4). **I** Serum levels of S-100B. **J** Plasma levels of FD-4. **K** Serum levels of LPS. **I**–**K**, n = 6) All data are shown as mean ± SD. ^*^P < 0.05, ^**^P < 0.01, ^***^P < 0.001

ILA, as a ligand for AhR, has been confirmed to maintain the intestinal epithelial barrier and modulate immune function, with efficacy in mitigating intestinal ischemia–reperfusion injury, ulcerative colitis, and colon cancer [42, 55, 56]. Previous studies have found that ILA has been shown to be neuroprotective in vitro, but no in vivo experiments have been performed [57]. We test our hypothesis by giving orally ILA to rats with ischemic stroke for 14 days. After 14 days of treatment, the body weight gain recovered substantially in the ILA group (Fig. 7A). Both the Longa score and the mNSS score decreased with time after ILA administration (Fig. 7B, C). Although there was no significant difference in survival curves, the survival rate of ILA was higher than that of MCAO (Fig. 7D). TTC staining revealed that significantly reduced infarct size was observed in the ILA group (Fig. 7E, F). As shown by Nissl and HE staining, ILA reversed the reduction of Nissl-positive cells as well as the reduction of histopathologic changes in the brain (Fig. 7G, H). Notably, reduced serum levels of S100B indicated that ILA repaired the blood–brain barrier (Fig. 7I). Goblet cell reduction was improved by ILA treatment (Fig. 7G). Furthermore, the ILA group exhibited considerably lower plasma FD-4 and serum LPS levels (Fig. 7J, K), which suggests that ILA may impede bacterial translocation and reduce intestinal permeability. To sum up, oral ILA treatment may prevent tissue and nerve damage in ischemic stroke rats while also reversing the disruption of the gut barrier brought on by the ischemic stroke.

## Discussion

In the present study, we explored the efficacy and mechanism of BHTD in ischemic stroke. The results demonstrated that BHTD could mitigate ischemic stroke-induced brain and gut injury. Furthermore, BHTD reversed gut microbiota dysbiosis and upregulated tryptophan metabolism to enhance the synthesis of ILA thereby mitigating ischemic stroke. Our study provided evidence that traditional Chinese medicine formulas could treat central diseases by regulating intestinal homeostasis and established a scientific foundation for the therapeutic use of BHTD.

BHTD is a new formula prescribed by prestigious Chinese physician Professor Kang Peng. According to the *Chinese Pharmacopoeia*, Astragali Radix, Chuanxiong Rhizoma, Salviae Miltiorrhizae Radix et Rhizoma and Notoginseng Radix et Rhizoma are targeted at “Qi deficiency and blood stasis”. Paeoniae radix rubra, Pinelliae Rhizoma Praeparatum, Poria and Glycyrrhizae Radix et Rhizoma Praeparata Cum Melle, modified from Er-Chen Decoction, a Chinese traditional formula for resolving phlegm, was applied in the treatment of “phlegm-damp clouding orifices” and added with Pheretima for dredging collaterals [58]. Notably, Puerariae Lobatae Radix, which is rich in various flavonoids, such as puerarin, daidzin and daidzein, can increase blood flow to the brain and coronary blood vessels to improve cerebral ischemia [15]. Moreover, our previous studies have demonstrated that Puerariae Lobatae Radix-resistant starch, an insoluble macromolecule, has excellent protective effects against ischemic stroke through a favorable microbial co-occurrence pattern [29]. In conclusion, BHTD generates effects by “invigorating qi and promoting blood circulation, dissipating phlegm and dredging brain”, which is regarded as improving apoplexy targeting by its pathogenesis. In our study, BHTD demonstrated efficacy in reducing central damage, mitigating intestinal injury and regulating lipid metabolism, while Nimodipine, a calcium channel blocker, only showed the effect of decreasing central injury, which was attributed to its single-target action. Therefore, BHTD synergized the effects through multi-pathway, multi-target, and holistic modulation, which are worthy of further application and promotion in clinical practice.

Decoction is the most commonly utilized preparation form of TCM, which enters the body orally and therefore impacts the gut microbiota in the gastrointestinal tract. In the present study, the gut microbiota of ischemic stroke rats was shown to be enriched with a large number of pathogenic and conditionally pathogenic bacteria, including *Corynebacterium*, *Dielma* and *Escherichia-Shigella*. In addition, increased intestinal permeability and bacterial translocation demonstrated impaired gut barrier in ischemic stroke rats. These suggested that ischemic stroke-induced harmful bacteria and their metabolites entered the circulation and thus promoted systemic inflammation, which caused gastrointestinal complications and exacerbated the disease process. However, these intestinal pathologies can be reversed with BHTD. BHTD significantly enriched a variety of beneficial bacteria, especially *Turicibacter* and *Faecalibaculum*. *Turicibacter* is a prominent member of the mammalian gut microbiota [59]. Lin et al. have proved that *Turicibacter* fermentation could inhibit the Wnt pathway in Caco-2 colon cancer cells, suggesting its effectiveness on intestinal homeostasis [60]. *Turicibacter* could induce the gut to produce more serotonin, a neurotransmitter generated from the gut, thereby influencing brain function including cognition, learning and memory [61, 62]. *Faecalibaculum* has the potential to regulate intestinal epithelial homeostasis [63]. Notably, *Faecalibaculum* could produce rich influence on hippocampus synaptic plasticity and neurotransmission, which in turn affects spatial learning and memory [64, 65]. Spearman’s correlation analysis suggested that the abundance of *Turicibacter* and *Faecalibaculum* was positively related to the indicators of ischemic stroke recovery, containing traits of cerebral damage, traits of intestinal barrier and risk factors for thrombosis. Therefore, we speculated that the gut microbiota remodeled by BHTD, especially the enrichment of *Turicibacter* and *Faecalibaculum*, was an upstream target for its protective effects. Then, we performed fecal microbiota transplantation experiments and proved that transplantation of BHTD-regulated microbiota into ischemic stroke rats recapitulated the cerebral and intestinal protective effects of BHTD, which confirmed our speculations. However, the effects of *Turicibacter* and *Faecalibaculum* on ischemic stroke require further study.

Changes in the composition of the gut microbiota are always accompanied by alterations in metabolism [66]. We found that the metabolite profiles of the MCAO and BHTD-H groups differed significantly. KEGG pathway enrichment analysis showed 10 pathways changed, such as “glycerophospholipid metabolism”, “glycine, serine and threonine metabolism”, “primary bile acid biosynthesis” and “tryptophan metabolism”. Particularly, we observed an upregulation of the tryptophan metabolism pathway, which is intimately linked to the ischemic stroke process and is consistent with the findings of the microbial PICRUSt2 research. Tryptophan is an essential amino acid that can’t be produced by animal cells and therefore needs to be consumed through diet [67]. Tryptophan metabolism in the body follows three major pathways [68]. When absorbed into the host, on the one hand, tryptophan is metabolized by tryptophan hydroxylase 1 (TpH1) to serotonin, a neurotransmitter involved in the regulation of mood control, sleep, and pain processing; on the other hand, most tryptophan is metabolized in the liver via indoleamine 2,3-dioxygenase (IDO) 1 into kynurenine and its derivatives, which can cross the BBB and modulate various brain and gastrointestinal functions [69]. However, tryptophan metabolites in the two pathways were absent in the screened differential metabolites in our study. Given that unabsorbed tryptophan would reach the colon, where it is metabolized into indole and its derivatives through the gut microbiota; this is known as the indole pathway of tryptophan metabolism. We found that indoles were altered after the intervention of BHTD, containing indole lactic acid (ILA), indole acetamide and indole propionic acid. Among them, ILA was significantly upregulated. Furthermore, the targeted metabolomics results confirmed that the level of ILA in cecum contents was significantly higher in the BHTD-H group than in the MCAO group. Likewise, we performed in vitro fermentation experiments, which directly demonstrated that ILA was derived from gut microbiota and that BHTD could remodel gut microbiota in ischemic stroke rats to produce higher levels of ILA. ILA has been proven to be produced by *Lactobacillus, Bifidobacterium* and *Escherichia coli* [70–72]. Specifically speaking, *Lactobacillus* species could produce ILA (*Lactobacillus plantarum* [31, 73], *Lacticaseibacillus paracasei* [74], and *Lactobacillus salivarius* [75]; human gut-associated *Bifidobacterium* species could synthesize ILA (*Bifidobacterium breve* [76], *Bifidobacterium bifidum* [55] and infant-type Human-Residential *Bifidobacteria* [32]); *Escherichia coli* have been proven to promote ILA (*Ec*-TMU [72] and an engineered *Escherichia coli* Nissle 1917 [77, 78]). Interestingly, LEfSe analysis showed that *Lactobacillus* was enriched in the BHTD-H group and the relative abundance of *Lactobacillus* in the BHTD-H group is significantly higher than in the MCAO group. Therefore, we speculate that this might account for the increased levels of ILA. However, whether the screened biomarkers, *Turicibacter* and *Faecalibaculum*, can also produce ILA remains to be further investigated.

ILA as a ligand for AHR, could act on epithelium renewal and modulate immune cells to help maintain intestinal homeostasis [79, 80]: ILA promotes the self-renewal of intestinal epithelial cells through YAP regulation. [35]; ILA also inhibits tumor growth by improving CD8^+^ T cell activity [31]. Moreover, ILA alleviates neuro-degeneration in HT-22 cells and enhances neurite growth in PC 12 cells, indicating that ILA has a promising effect on modulating the gut and the brain [32, 81]. Therefore, we speculated that the efficacy of BHTD in repairing the brain and gut was partially attributable to ILA produced by the gut microbiota. Gavage of ILA reduced the loss of goblet cells and the thinning of the mucosal layer in the colon of ischemic stroke rats, which was consistent with enteroprotective effects of ILA reported previously. Previous studies on the neural effects of ILA rested on in vitro experiments, so we explored the protective effects of ILA on the brain in vivo first. Administration of ILA by gavage effectively reduced the extent of cerebral infarction as well as ameliorated neurological damage in ischemic stroke rats. Moreover, we examined the levels of ILA in serum and brain and found that the BHTD-H group had much larger levels than the MCAO group, which was consistent with the trend of cecal contents. These results suggested that ILA, a small molecule produced by the gut microbiota, could eventually be enriched in the brain by passing through the blood–brain and intestinal barriers. Our findings confirm ILA as a novel target for the treatment of ischemic stroke and provide new insights for more applications of ILA.

## Conclusion

**Fig. 8:**
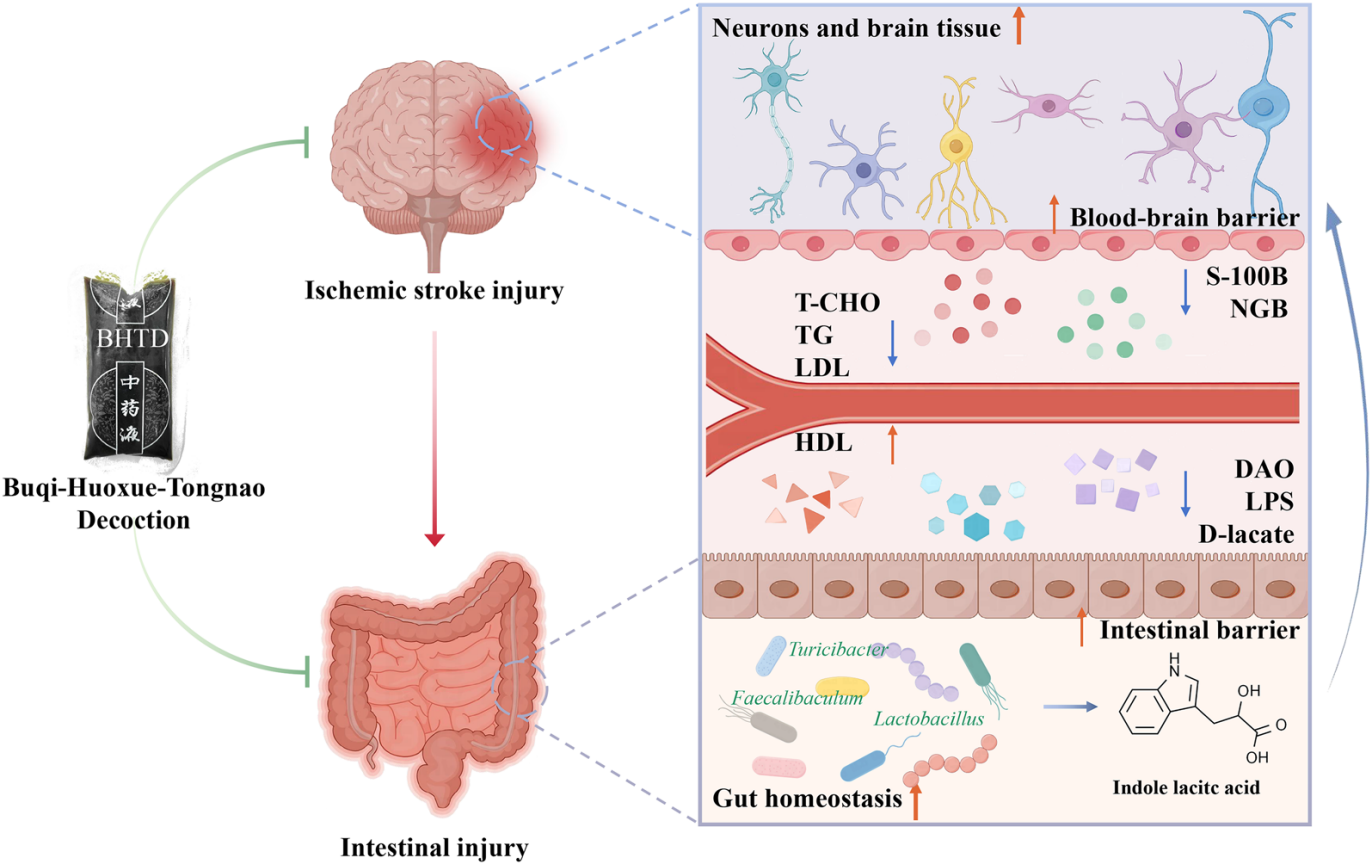
Buqi-Huoxue-Tongnao decoction drives gut microbiota-derived indole lactic acid to attenuate ischemic stroke via the gut-brain axis

BHTD could remodel the gut microbiota to upregulate tryptophan metabolism, thus driving indole lactic acid derived from the gut microbiota to attenuate ischemic stroke (Fig. 8). Our current finding provides evidence that traditional Chinese medicine treats ischemic stroke via the gut-brain axis.

## Supplementary Information

Supplementary material 1

## Data Availability

The data that support the findings of this study are available from the corresponding author upon reasonable request.

## Abbreviations

BHTD: Buqi-Huoxue-Tongnao decoction
IS: Ischemic stroke
TCM: Traditional Chinese medicine
ILA: Indole lactic acid
LPS: Lipopolysaccharides
SCFAs: Short-chain fatty acids
AHR: Aryl hydrocarbon receptor
MCAO: Middle cerebral artery occlusion
mNSS: Modified neurologic severity score
TTC: 2, 3, 5-Triphenyltetrazolium chloride
T-CHO: Total cholesterol
TG: Triglyceride
LDL: Low-density lipoprotein
HDL: High-density lipoprotein
BBB: Blood–brain barrier
TJPs: Tight junction proteins
NGB: Neuroglobin
FD-4: Fluorescein isothiocyanate dextran-4 kDa
DAO: Diamine oxidase
OTU: Operational taxonomic unit
PCoA: Principal coordinate analysis
NMDS: Non-metric multidimensional scaling
LEfSe: Linear discriminant analysis effect size
RDA: Redundancy analysis
PICRUSt: Phylogenetic investigation of communities by reconstruction of unobserved states
KEGG: Kyoto encyclopedia of genes and genome
FMT: Fecal microbiota transplantation
OPLS-DA: Orthogonal partial least squares discrimination analysis
VIP: Variable importance projection

## References

[1] Qin C, Yang S, Chu Y-H, Zhang H, Pang X-W, Chen L, et al. Signaling pathways involved in ischemic stroke: molecular mechanisms and therapeutic interventions. Sig Transduct Target Ther. 2022;7:1–29. 10.1038/s41392-022-01064-1 PMC9259607 35794095

[2] Shehjar F, Maktabi B, Rahman ZA, Bahader GA, James AW, Naqvi A, et al. Stroke: molecular mechanisms and therapies: Update on recent developments. Neurochem Int. 2023;162: 105458. 36460240 10.1016/j.neuint.2022.105458 PMC9839659

[3] Huang Y, Wang Z, Huang Z-X, Liu Z. Biomarkers and the outcomes of ischemic stroke. Front Mol Neurosci. 2023. 10.3389/fnmol.2023.1171101. 37342100 10.3389/fnmol.2023.1171101 PMC10277488

[4] Xiao A, Zhang Y, Ren Y, Chen R, Li T, You C, et al. GDF11 alleviates secondary brain injury after intracerebral hemorrhage via attenuating mitochondrial dynamic abnormality and dysfunction. Sci Rep. 2021;11:3974. 33597668 10.1038/s41598-021-83545-x PMC7889617

[5] Wang P-Q, Wu Y, Zhang M, Huo H-R, Du X-L, Sui F. Analysis of protective effects of Chaihu Jia Longgu Muli Tang against ischemic stroke by combining traditional Chinese medicine pathogenesis and efficacy with modern pathology and pharmacology. Zhongguo Zhong Yao Za Zhi. 2018;43:2448–53. 29950058 10.19540/j.cnki.cjcmm.2018.0076

[6] Song J, Xu C, Zhang J, Gao L. From clinical appearance to accurate management in acute ischemic stroke patients: with the guidance of innovative traditional Chinese medicine diagnosis. Brain and Behavior. 2019;9: e01411. 31566916 10.1002/brb3.1411 PMC6790312

[7] Luan X, Zhang L-J, Li X-Q, Rahman K, Zhang H, Chen H-Z, et al. Compound-based Chinese medicine formula: from discovery to compatibility mechanism. J Ethnopharmacol. 2020;254: 112687. 32105748 10.1016/j.jep.2020.112687

[8] Li J, Yu J, Ma H, Yang N, Li L, Zheng D, et al. Intranasal pretreatment with Z-Ligustilide, the main volatile component of rhizoma chuanxiong, confers prophylaxis against cerebral ischemia via Nrf2 and HSP70 signaling pathways. J Agric Food Chem. 2017;65:1533–42. 28169530 10.1021/acs.jafc.6b04979

[9] Shi Y-H, Zhang X-L, Ying P-J, Wu Z-Q, Lin L-L, Chen W, et al. Neuroprotective effect of astragaloside IV on cerebral ischemia/reperfusion injury rats through sirt1/mapt pathway. Front Pharmacol. 2021. 10.3389/fphar.2021.639898/full. 33841157 10.3389/fphar.2021.639898/full PMC8033022

[10] Guo W, Xu X, Xiao Y, Zhang J, Shen P, Lu X, et al. Salvianolic acid C attenuates cerebral ischemic injury through inhibiting neuroinflammation via the TLR4-TREM1-NF-κB pathway. Chin Med. 2024;19:46. 38468280 10.1186/s13020-024-00914-0 PMC10929175

[11] Zhao F, Peng C, Li H, Chen H, Yang Y, Ai Q, et al. *Paeoniae Radix Rubra* extract attenuates cerebral ischemia injury by inhibiting ferroptosis and activating autophagy through the PI3K/Akt signalling pathway. J Ethnopharmacol. 2023;315: 116567. 37172921 10.1016/j.jep.2023.116567

[12] He L, Chen X, Zhou M, Zhang D, Yang J, Yang M, et al. Radix/rhizoma notoginseng extract (Sanchitongtshu) for ischemic stroke: a randomized controlled study. Phytomedicine. 2011;18:437–42. 21094030 10.1016/j.phymed.2010.10.004

[13] Sun Z-Y, Wang F-J, Guo H, Chen L, Chai L-J, Li R-L, et al. Shuxuetong injection protects cerebral microvascular endothelial cells against oxygen-glucose deprivation reperfusion. Neural Regen Res. 2019;14:783. 30688264 10.4103/1673-5374.249226 PMC6375046

[14] Zheng M, Zhou M. Neuroprotective effect of daidzein extracted from pueraria lobate radix in a stroke model via the Akt/mTOR/BDNF channel. Front Pharmacol. 2022. 10.3389/fphar.2021.772485/full. 35095491 10.3389/fphar.2021.772485/full PMC8795828

[15] Zhou Y-X, Zhang H, Peng C. Puerarin: a review of pharmacological effects. Phytother Res. 2014;28:961–75. 24339367 10.1002/ptr.5083

[16] Peng K, Zhu Z, Cai Z, Ma K, Luo L, Liu M, et al. Traditional Chinese medicine composition, preparation method thereof and application of traditional Chinese medicine composition in preparation of medicine for treating or preventing chronic cerebral blood supply insufficiency. 2023.

[17] Ma Z, Jia C, Guo J, Gu H, Miao Y. Features analysis of five-element theory and its basal effects on construction of visceral manifestation theory. J Tradit Chin Med. 2014;34:115–21. 25102701 10.1016/S0254-6272(14)60064-9

[18] Zhao Y, Zhuang Y. Modern interpretation of “exterior-interior correlation between lung and large intestine” theory in acute and critical cases. Zhonghua Wei Zhong Bing Ji Jiu Yi Xue. 2020;32:1040–4. 33081887 10.3760/cma.j.cn121430-20200603-00792

[19] Han Z-T. Brain governing mind theory in traditional chinese medicine. Zhonghua Yi Shi Za Zhi. 2012;42:195–8. 23336273

[20] Ge Y, Wang X, Guo Y, Yan J, Abuduwaili A, Aximujiang K, et al. Gut microbiota influence tumor development and Alter interactions with the human immune system. J Exp Clin Cancer Res. 2021;40:42. 33494784 10.1186/s13046-021-01845-6 PMC7829621

[21] Camara-Lemarroy CR, Ibarra-Yruegas BE, Gongora-Rivera F. Gastrointestinal complications after ischemic stroke. J Neurol Sci. 2014;346:20–5. 25214444 10.1016/j.jns.2014.08.027

[22] Chidambaram SB, Rathipriya AG, Mahalakshmi AM, Sharma S, Hediyal TA, Ray B, et al. The influence of gut dysbiosis in the pathogenesis and management of ischemic stroke. Cells. 2022;11:1239. 35406804 10.3390/cells11071239 PMC8997586

[23] Xu K, Gao X, Xia G, Chen M, Zeng N, Wang S, et al. Rapid gut dysbiosis induced by stroke exacerbates brain infarction in turn. Gut. 2021;70:1486–94. 10.1136/gutjnl-2020-323263 33558272

[24] Rahman Z, Bhale NA, Dikundwar AG. Multistrain probiotics with fructooligosaccharides improve middle cerebral artery occlusion-driven neurological deficits by revamping microbiota-gut-brain axis. Probiot Antimicro Prot. 2023. 10.1007/s12602-023-10109-y. 10.1007/s12602-023-10109-y 37365420

[25] Wang Q, Yang Q, Liu X. The microbiota–gut–brain axis and neurodevelopmental disorders. Protein Cell. 2023;14:762–75. 37166201 10.1093/procel/pwad026 PMC10599644

[26] Liu L, Huh JR, Shah K. Microbiota and the gut-brain-axis: Implications for new therapeutic design in the CNS. eBioMedicine. 2022;77:103908. 35255456 10.1016/j.ebiom.2022.103908 PMC8897630

[27] Brown EM, Clardy J, Xavier RJ. Gut microbiome lipid metabolism and its impact on host physiology. Cell Host Microbe. 2023;31:173–86. 36758518 10.1016/j.chom.2023.01.009 PMC10124142

[28] Brouns R, Verkerk R, Aerts T, De Surgeloose D, Wauters A, Scharpé S, et al. The role of tryptophan catabolism along the kynurenine pathway in acute ischemic stroke. Neurochem Res. 2010;35:1315–22. 20490917 10.1007/s11064-010-0187-2

[29] Lian Z, Xu Y, Wang C, Chen Y, Yuan L, Liu Z, et al. Gut microbiota-derived melatonin from *Puerariae**Lobatae* radix-resistant starch supplementation attenuates ischemic stroke injury via a positive microbial co-occurrence pattern. Pharmacol Res. 2023;190: 106714. 36863429 10.1016/j.phrs.2023.106714

[30] Zhou Y, Chen Y, He H, Peng M, Zeng M, Sun H. The role of the indoles in microbiota-gut-brain axis and potential therapeutic targets: a focus on human neurological and neuropsychiatric diseases. Neuropharmacology. 2023;239: 109690. 37619773 10.1016/j.neuropharm.2023.109690

[31] Zhang Q, Zhao Q, Li T, Lu L, Wang F, Zhang H, et al. Lactobacillus plantarum-derived indole-3-lactic acid ameliorates colorectal tumorigenesis via epigenetic regulation of CD8+ T cell immunity. Cell Metab. 2023;35:943-960.e9. 37192617 10.1016/j.cmet.2023.04.015

[32] Wong CB, Tanaka A, Kuhara T, Xiao J. Potential effects of indole-3-lactic acid, a metabolite of human bifidobacteria, on ngf-induced neurite outgrowth in PC12 cells. Microorganisms. 2020;8:398. 32178456 10.3390/microorganisms8030398 PMC7143819

[33] Wang Y, Wang X, Li Y, Xue Z, Shao R, Li L, et al. Xuanfei Baidu Decoction reduces acute lung injury by regulating infiltration of neutrophils and macrophages via PD-1/IL17A pathway. Pharmacol Res. 2022;176: 106083. 35033647 10.1016/j.phrs.2022.106083 PMC8757644

[34] Longa EZ, Weinstein PR, Carlson S, Cummins R. Reversible middle cerebral artery occlusion without craniectomy in rats. Stroke. 1989;20:84–91. 2643202 10.1161/01.STR.20.1.84

[35] Zhang F-L, Chen X-W, Wang Y-F, Hu Z, Zhang W-J, Zhou B-W, et al. Microbiota-derived tryptophan metabolites indole-3-lactic acid is associated with intestinal ischemia/reperfusion injury via positive regulation of YAP and Nrf2. J Transl Med. 2023;21:264. 37072757 10.1186/s12967-023-04109-3 PMC10111656

[36] Chen J, Sanberg PR, Li Y, Wang L, Lu M, Willing AE, et al. Intravenous administration of human umbilical cord blood reduces behavioral deficits after stroke in rats. Stroke. 2001;32:2682–8. 11692034 10.1161/hs1101.098367

[37] Ding W, Cai C, Zhu X, Wang J, Jiang Q. Parthenolide ameliorates neurological deficits and neuroinflammation in mice with traumatic brain injury by suppressing STAT3/NF-κB and inflammasome activation. Int Immunopharmacol. 2022;108: 108913. 35729839 10.1016/j.intimp.2022.108913

[38] Swanson RA, Morton MT, Tsao-Wu G, Savalos RA, Davidson C, Sharp FR. A Semiautomated method for measuring brain infarct volume. J Cereb Blood Flow Metab. 1990;10:290–3. 1689322 10.1038/jcbfm.1990.47

[39] Volynets V, Reichold A, Bárdos G, Rings A, Bleich A, Bischoff SC. Assessment of the intestinal barrier with five different permeability tests in healthy C57BL/6J and BALB/cJ mice. Dig Dis Sci. 2016;61:737–46. 26520109 10.1007/s10620-015-3935-y

[40] You Y, Liang D, Wei R, Li M, Li Y, Wang J, et al. Evaluation of metabolite-microbe correlation detection methods. Anal Biochem. 2019;567:106–11. 30557528 10.1016/j.ab.2018.12.008

[41] Tintelnot J, Xu Y, Lesker TR, Schönlein M, Konczalla L, Giannou AD, et al. Microbiota-derived 3-IAA influences chemotherapy efficacy in pancreatic cancer. Nature. 2023;615:168–74. 36813961 10.1038/s41586-023-05728-y PMC9977685

[42] Li D, Feng Y, Tian M, Ji J, Hu X, Chen F. Gut microbiota-derived inosine from dietary barley leaf supplementation attenuates colitis through PPARγ signaling activation. Microbiome. 2021;9:83. 33820558 10.1186/s40168-021-01028-7 PMC8022418

[43] Zhou Y, Feng Y, Cen R, Hou X, Yu H, Sun J, et al. San-Wu-Huang-Qin decoction attenuates tumorigenesis and mucosal barrier impairment in the AOM/DSS model by targeting gut microbiome. Phytomedicine. 2022;98: 153966. 35158238 10.1016/j.phymed.2022.153966

[44] Tziomalos K, Athyros VG, Karagiannis A, Mikhailidis DP. Dyslipidemia as a risk factor for ischemic stroke. Curr Top Med Chem. 2009;9:1291–7. 19849661 10.2174/156802609789869628

[45] Abbott NJ, Patabendige AAK, Dolman DEM, Yusof SR, Begley DJ. Structure and function of the blood–brain barrier. Neurobiol Dis. 2010;37:13–25. 19664713 10.1016/j.nbd.2009.07.030

[46] Kadry H, Noorani B, Cucullo L. A blood–brain barrier overview on structure, function, impairment, and biomarkers of integrity. Fluids Barriers CNS. 2020;17:69. 33208141 10.1186/s12987-020-00230-3 PMC7672931

[47] Koh SXT, Lee JKW. S100B as a marker for brain damage and blood-brain barrier disruption following exercise. Sports Med. 2014;44:369–85. 24194479 10.1007/s40279-013-0119-9

[48] Yu Z, Liu N, Liu J, Yang K, Wang X. Neuroglobin, a novel target for endogenous neuroprotection against stroke and neurodegenerative disorders. Int J Mol Sci. 2012;13:6995–7014. 22837676 10.3390/ijms13066995 PMC3397508

[49] Park S-Y, Kim J, Kim O-J, Kim J-K, Song J, Shin D-A, et al. Predictive value of circulating interleukin-6 and heart-type fatty acid binding protein for three months clinical outcome in acute cerebral infarction: multiple blood markers profiling study. Crit Care. 2013;17:R45. 23497639 10.1186/cc12564 PMC3672476

[50] Ge Y, Zadeh M, Yang C, Candelario-Jalil E, Mohamadzadeh M. Ischemic stroke impacts the gut microbiome. Ileal Epithelial Immune Homeostasis iSci. 2022;25: 105437. 10.1016/j.isci.2022.105437 PMC9650036 36388972

[51] Camilleri M. Leaky gut: mechanisms, measurement and clinical implications in humans. Gut. 2019;68:1516–26. 31076401 10.1136/gutjnl-2019-318427 PMC6790068

[52] Adelman MW, Woodworth MH, Langelier C, Busch LM, Kempker JA, Kraft CS, et al. The gut microbiome’s role in the development, maintenance, and outcomes of sepsis. Crit Care. 2020;24:278. 32487252 10.1186/s13054-020-02989-1 PMC7266132

[53] Zhang F, Aschenbrenner D, Yoo JY, Zuo T. The gut mycobiome in health, disease, and clinical applications in association with the gut bacterial microbiome assembly. Lancet Microbe. 2022;3:e969–83. 36182668 10.1016/S2666-5247(22)00203-8

[54] Huang Y, Zhao M, Chen X, Zhang R, Le A, Hong M, et al. Tryptophan metabolism in central nervous system diseases: pathophysiology and potential therapeutic strategies. Aging Dis. 2023;14:858–78. 37191427 10.14336/AD.2022.0916 PMC10187711

[55] Cui Q, Zhang Z, Tian X, Liang X, Lu Y, Shi Y, et al. Bifidobacterium bifidum ameliorates DSS-induced colitis in mice by regulating AHR/NRF2/NLRP3 inflammasome pathways through indole-3-lactic acid production. J Agric Food Chem. 2023;71:1970–81. 36633059 10.1021/acs.jafc.2c06894

[56] Sugimura N, Li Q, Chu ESH, Lau HCH, Fong W, Liu W, et al. *Lactobacillus**gallinarum* modulates the gut microbiota and produces anti-cancer metabolites to protect against colorectal tumourigenesis. Gut. 2022;71:2011–21. 10.1136/gutjnl-2020-323951 PMC9484392 34937766

[57] Serger E, Luengo-Gutierrez L, Chadwick JS, Kong G, Zhou L, Crawford G, et al. The gut metabolite indole-3 propionate promotes nerve regeneration and repair. Nature. 2022;607:585–92. 35732737 10.1038/s41586-022-04884-x

[58] Chen J, Ye C, Yang Z, Zhang C, Li P, Xu B, et al. Erchen decoction to reduce oxidative stress in dyslipidemia phlegm-dampness retention syndrome mice: In vivo mechanism revealed by metabolomics (liquid chromatography–mass spectrometry). Phytomedicine. 2023;115: 154808. 37087794 10.1016/j.phymed.2023.154808

[59] Lynch JB, Gonzalez EL, Choy K, Faull KF, Jewell T, Arellano A, et al. Gut microbiota Turicibacter strains differentially modify bile acids and host lipids. Nat Commun. 2023;14:3669. 37339963 10.1038/s41467-023-39403-7 PMC10281990

[60] Lin T-C, Soorneedi A, Guan Y, Tang Y, Shi E, Moore MD, et al. Turicibacter fermentation enhances the inhibitory effects of *Antrodia**camphorata* supplementation on tumorigenic serotonin and Wnt pathways and promotes ROS-mediated apoptosis of Caco-2 cells. Front Pharmacol. 2023. 10.3389/fphar.2023.1203087. 37663253 10.3389/fphar.2023.1203087 PMC10469317

[61] Yang Y, Lu M, Xu Y, Qian J, Le G, Xie Y. Dietary methionine via dose-dependent inhibition of short-chain fatty acid production capacity contributed to a potential risk of cognitive dysfunction in mice. J Agric Food Chem. 2022;70:15225–43. 36413479 10.1021/acs.jafc.2c04847

[62] Huang L, Liu Z, Wu P, Yue X, Lian Z, He P, et al. *Puerariae**Lobatae* radix alleviates pre-eclampsia by remodeling gut microbiota and protecting the gut and placental barriers. Nutrients. 2022;14:5025. 36501055 10.3390/nu14235025 PMC9738998

[63] Cao YG, Bae S, Villarreal J, Moy M, Chun E, Michaud M, et al. *Faecalibaculum**rodentium* remodels retinoic acid signaling to govern eosinophil-dependent intestinal epithelial homeostasis. Cell Host Microbe. 2022;30:1295-1310.e8. 35985335 10.1016/j.chom.2022.07.015 PMC9481734

[64] Zagato E, Pozzi C, Bertocchi A, Schioppa T, Saccheri F, Guglietta S, et al. Endogenous murine microbiota member Faecalibaculum rodentium and its human homologue protect from intestinal tumour growth. Nat Microbiol. 2020;5:511–24. 31988379 10.1038/s41564-019-0649-5 PMC7048616

[65] D’Amato A, Di Cesare ML, Lucarini E, Man AL, Le Gall G, Branca JJV, et al. Faecal microbiota transplant from aged donor mice affects spatial learning and memory via modulating hippocampal synaptic plasticity- and neurotransmission-related proteins in young recipients. Microbiome. 2020;8:140. 33004079 10.1186/s40168-020-00914-w PMC7532115

[66] Tang WHW, Li DY, Hazen SL. Dietary metabolism, the gut microbiome, and heart failure. Nat Rev Cardiol. 2019;16:137–54. 30410105 10.1038/s41569-018-0108-7 PMC6377322

[67] Gao K, Mu C, Farzi A, Zhu W. Tryptophan metabolism: a link between the gut microbiota and brain. Adv Nutr. 2020;11:709–23. 31825083 10.1093/advances/nmz127 PMC7231603

[68] Roth W, Zadeh K, Vekariya R, Ge Y, Mohamadzadeh M. Tryptophan metabolism and gut-brain homeostasis. Int J Mol Sci. 2021;22:2973. 33804088 10.3390/ijms22062973 PMC8000752

[69] Correia AS, Vale N. Tryptophan metabolism in depression: a narrative review with a focus on serotonin and kynurenine pathways. Int J Mol Sci. 2022;23:8493. 35955633 10.3390/ijms23158493 PMC9369076

[70] Miao H, Wang Y-N, Yu X-Y, Zou L, Guo Y, Su W, et al. Lactobacillus species ameliorate membranous nephropathy through inhibiting the aryl hydrocarbon receptor pathway via tryptophan-produced indole metabolites. Br J Pharmacol. 2024;181:162–79. 37594378 10.1111/bph.16219

[71] Yong CC, Sakurai T, Kaneko H, Horigome A, Mitsuyama E, Nakajima A, et al. Human gut-associated Bifidobacterium species salvage exogenous indole, a uremic toxin precursor, to synthesize indole-3-lactic acid via tryptophan. Gut Microbes. 2024;16:2347728. 38706226 10.1080/19490976.2024.2347728 PMC11085991

[72] Yu K, Li Q, Sun X, Peng X, Tang Q, Chu H, et al. Bacterial indole-3-lactic acid affects epithelium-macrophage crosstalk to regulate intestinal homeostasis. Proc Natl Acad Sci USA. 2023;120: e2309032120. 37903267 10.1073/pnas.2309032120 PMC10636326

[73] Zhou Q, Xie Z, Wu D, Liu L, Shi Y, Li P, et al. The effect of indole-3-lactic acid from *Lactiplantibacillus**plantarum* ZJ316 on human intestinal microbiota in vitro. Foods. 2022;11:3302. 37431049 10.3390/foods11203302 PMC9601829

[74] Yao M, Cao J, Zhang L, Wang K, Lin H, Qin L, et al. Indole-3-lactic acid derived from *Lacticaseibacillus**paracasei* inhibits helicobacter pylori infection via destruction of bacteria cells, protection of gastric mucosa epithelial cells, and alleviation of inflammation. J Agric Food Chem. 2024;72:15725–39. 38973111 10.1021/acs.jafc.4c02868

[75] Xia J, Jiang S, Lv L, Wu W, Wang Q, Xu Q, et al. Modulation of the immune response and metabolism in germ-free rats colonized by the probiotic *Lactobacillus**salivarius* LI01. Appl Microbiol Biotechnol. 2021;105:1629–45. 33507355 10.1007/s00253-021-11099-z

[76] Li Y, Li Q, Yuan R, Wang Y, Guo C, Wang L. Bifidobacterium breve-derived indole-3-lactic acid ameliorates colitis-associated tumorigenesis by directing the differentiation of immature colonic macrophages. Theranostics. 2024;14:2719–35. 38773969 10.7150/thno.92350 PMC11103503

[77] Dimopoulou C, Bongers M, Pedersen M, Bahl MI, Sommer MOA, Laursen MF, et al. An engineered *Escherichia**coli* Nissle 1917 increase the production of indole lactic acid in the gut. FEMS Microbiol Lett. 2023;370:027. 10.1093/femsle/fnad027 37028942

[78] Dimopoulou C, Guerra PR, Mortensen MS, Kristensen KA, Pedersen M, Bahl MI, et al. Potential of using an engineered indole lactic acid producing *Escherichia**coli* Nissle 1917 in a murine model of colitis. Sci Rep. 2024;14:17542. 39080343 10.1038/s41598-024-68412-9 PMC11289411

[79] Busbee PB, Menzel L, Alrafas HR, Dopkins N, Becker W, Miranda K, et al. Indole-3-carbinol prevents colitis and associated microbial dysbiosis in an IL-22–dependent manner. JCI Insight. 2020. 5. https://insight.jci.org/articles/view/127551 10.1172/jci.insight.127551 PMC7030851 31941837

[80] Aoki R, Aoki-Yoshida A, Suzuki C, Takayama Y. Indole-3-pyruvic acid, an Aryl hydrocarbon receptor activator, suppresses experimental colitis in mice. J Immunol. 2018;201:3683–93. 30429284 10.4049/jimmunol.1701734

[81] Yin J, Zhang Y, Liu X, Li W, Hu Y, Zhang B, et al. Gut microbiota-derived indole derivatives alleviate neurodegeneration in aging through activating GPR30/AMPK/SIRT1 pathway. Mol Nutr Food Res. 2023;67:2200739. 10.1002/mnfr.202200739 36823436

