# Supplementary material for: Buqi-Huoxue-Tongnao decoction drives gut microbiota-derived indole lactic acid to attenuate ischemic stroke via the gut-brain axis
Source: Chin Med. 2024 Sep 15;19:126. doi: 10.1186/s13020-024-00991-1 (PMC11403783; doi:10.1186/s13020-024-00991-1)
Supplement: Supplementary file 1 — Supplementary material 1 [file 13020_2024_991_MOESM1_ESM.docx]

**Supplementary Materials For**

**Buqi-Huoxue-Tongnao decoction drives gut microbiota-derived indole lactic acid to attenuate ischemic stroke via the gut-brain axis**

Yarui Liu^a,1^, Peng Zhao^b,1^, Zheng Cai^a,c^, Peishi He^a^, Jiahan Wang^a^, Haoqing He^a^, Zhibo Zhu^b^, Xiaowen Guo^b^, Ke Ma^b^, Kang Peng^b*^ and Jie Zhao^a,b,c,d*^

**This file includes:**

**Table S1.** Compositions of BHTD.

**Table S2.** The sample elution procedure.

**Table S3.** The criteria of mNSS score.

**Table S4.** Primer sequences for qPCR.

**Table S5.** Spearman's correlation analysis between the relative abundance of microbiota and efficacy parameters.

**Table S6.** The final altered metabolites

**Table S7.** Pathway analysis between the MCAO group and the BHTD-H group.

**Figure S1.** LC-MS/MS spectrums of BHTD.

**Figure S2.** Pearson correlation analyses of the levels of Claudin-1, Occludin and ZO-1 between the brain and colon.

**Figure S3.** The effect of BHTD supplementation on gut microbiota.

**Figure S4.** BHTD altered the gut microbial profiles and promoted the production of gut microbiota-derived indole lactic acid.

Table S1. Compositions of BHTD

| Botanical name | Chinese name | Species | Medicinal parts | Raw herb weight | Role in prescription |
| --- | --- | --- | --- | --- | --- |
| Astragali Radix | Huang Qi | *Astragalus mongholicus* Bunge | Dried root | 15g | Monarch |
| Chuanxiong Rhizoma | Chuan Xiong | *Ligusticum chuanxiong* Hort. | Dried root | 10g |  |
| Salviae Miltiorrhizae Radix et Rhizoma | Dan Shen | *Salvia miltiorrhiza* Bunge | Dried root | 10g | Minister |
| Notoginseng Radix et Rhizoma | San Qi | *Panax notoginseng* (Burkill) F.H.Chen | Dried root | 9g |  |
| Paeoniae radix rubra | Chi Shao | *Paeonia lactiflora* Pall. | Dried root | 12g |  |
| Pheretima | Di Long | *Pheretima aspergillum* (E.Perrier) | Dried body | 10g |  |
| Poria | Fu Ling | *Poria cocos* (Schw.) Wolf | Dried sclerotium | 15g | Assistant |
| Puerariae Lobatae Radix | Ge Gen | *Pueraria montana var. lobata* (Willd.) | Dried root | 15g |  |
| Pinelliae Rhizoma Praeparatum | Fa Ban Xia | *Pinellia ternata* (Thunb.) Makino | Dried tuber | 9g |  |
| Glycyrrhizae Radix et Rhizoma Praeparata Cum Melle | Zhi Gan Cao | *Glycyrrhiza uralensis* Fisch. | Dried root | 10g | Guide |

Table S2. The sample elution procedure.

| Time (min) | Flow (mL/min) | Phase B（%） |
| --- | --- | --- |
| 0 | 0.5 | 1 |
| 3 | 0.5 | 1 |
| 15 | 0.5 | 95 |
| 20 | 0.5 | 95 |
| 20.1 | 0.5 | 1 |

Samples were separated on a Thermo Hypersil Gold HPLC Column (100 x 2.1 mm) at the flow rate of 0.5 ml/min. Mobile phases A and B were water + 0.1% formic acid and acetonitrile + 0.1% formic acid. Injection Volume was 2uL and column temperature was 50°C. The high-resolution mass spectrometry analysis was mainly performed under the following parameters: Ion Source, H-ESI; Positive, 3800V; Sheath Gas (Arb), 35; Aux Gas (Arb), 8; Sweep Gas (Arb), 0; Ion Transfer Tube Temp, 275 °C; Vaporizer Temp, 200 °C; Scans, Full Scan –ddms2; Obitrap Resolution, 60000.

Table S3. The criteria of mNSS score

| Scoring content | | Scoring criteria | Score |
| --- | --- | --- | --- |
| Movement experiments | Tail lifting experiment | Forelimb flexion | 1 |
|  |  | Hind limb flexion | 1 |
|  |  | Head deviates from vertical axis within 30 s (>100°) | 1 |
|  | Walk to observe | Normal walking | 0 |
|  |  | Cannot walk in a straight line | 1 |
|  |  | Rotation to the lightly paralyzed side | 2 |
|  |  | Leaning toward the lightly paralyzed side | 3 |
| Sensory experiment | Placement experiment | Visual and tactile testing | 1 |
|  | Proprioception experiment | Deep sensation, stimulating limb muscles | 1 |
|  | Balance beam experiment | Stabilizing the balance beam | 0 |
|  |  | Clutching the edge of the balance beam | 1 |
|  |  | Clutching a balance beam, one limb falls off the beam | 2 |
|  |  | Holding a balance beam tightly, falling or spinning on both limbs from the beam (>60 s) | 3 |
|  |  | Trying to balance on a balance beam but falls (>40 s) | 4 |
|  |  | Trying to balance on a balance beam but falls (>20 s) | 5 |
|  |  | Falling: no attempt to balance on a balance beam (<20 s) | 6 |
| Reflex loss and abnormal movements | Auricular response | No shaking of the head when in contact with the external auditory canal | 1 |
|  | Corneal reaction | No blinking when cotton filaments lightly touch the cornea | 1 |
|  | Panic reaction | No motor response to the noise of fast-bouncing cardboard | 1 |
|  | Abnormal movement | Epilepsy, myoclonus, dystonia | 1 |

Table S4. Primer sequences for qPCR.

| Gene | Forward primers (3’-5’) | Reverse primers (5’-3’) |
| --- | --- | --- |
| *Claudin-1* | GGACACAAAGATTGCGATCAG | TCGACTCCTTGCTGAATCTG |
| *Occludin* | TCACTGTGTGACCTGTCTTGG | ACTGGGCTGGATGCCAATTT |
| *Zo-1* | ACAGCCAGCTCTTGGTCATC | GTATGGTGGCTGCTCAAGGT |
| *β-actin* | GGAGATTACTGCCCTGGCTCCTAGC | GGCCGGACTCATCGTACTCCTGCTT |

Table S5. Spearman's correlation analysis between the relative abundance of microbiota and efficacy parameters.

| **Correlation (r value)** | | | | | | | | | | | | | | | | | | | | | |
| --- | --- | --- | --- | --- | --- | --- | --- | --- | --- | --- | --- | --- | --- | --- | --- | --- | --- | --- | --- | --- | --- |
| **Name** | **Traits of cerebral injury** | | | | | | | | | | **Traits of intestinal barrier** | | | | | | | **Factors of thrombosis risk** | | | |
|  | **Relative body weight** | **Number of Nissl possitive cell** | **Longa Score** | **mNSS Score** | **S100B in serum** | **NGB in serum** | **Cerebral infarction volume** | **Claudin-1 in brain** | **Occludin in brain** | **ZO-1 in brain** | **Claudin-1 in colon** | **Occludin in colon** | **ZO-1 in colon** | **FD4 in plasma** | **DAO in serum** | **LPS in serum** | **D-Lactate in serum** | **T-CHO** | **TG** | **HDL** | **LDL** |
| g__Adlercreutzia | 0.1189 | 0.2007 | -0.3349 | -0.0072 | -0.2721 | -0.6364 | -0.1210 | 0.1748 | 0.1399 | 0.3328 | 0.1748 | 0.0877 | 0.2168 | -0.2751 | -0.0559 | -0.1399 | -0.1748 | -0.0666 | -0.0629 | 0.0350 | -0.0839 |
| g__Brachybacterium | -0.5783 | -0.6410 | 0.6333 | 0.6658 | 0.7064 | 0.4244 | 0.7157 | -0.5284 | -0.5409 | -0.5022 | -0.6657 | -0.5490 | -0.6823 | 0.7597 | 0.7031 | 0.6823 | 0.7572 | 0.6418 | 0.5700 | -0.6033 | 0.7281 |
| g__Collinsella | 0.6294 | 0.6549 | -0.5502 | -0.5578 | -0.7315 | -0.6434 | -0.5873 | 0.6713 | 0.6364 | 0.4098 | 0.6573 | 0.4807 | 0.5524 | -0.7372 | -0.4406 | -0.6713 | -0.6364 | -0.6130 | -0.5245 | 0.6084 | -0.5944 |
| g__Corynebacterium | -0.3077 | -0.4472 | 0.6020 | 0.4030 | 0.5760 | 0.8811 | 0.4520 | -0.3986 | -0.3007 | -0.4974 | -0.4056 | -0.3684 | -0.4965 | 0.4833 | 0.3706 | 0.4615 | 0.3636 | 0.5394 | 0.4336 | -0.3776 | 0.4965 |
| g__Dubosiella | 0.5464 | 0.4127 | -0.5831 | -0.5407 | -0.4744 | -0.7706 | -0.4421 | 0.4939 | 0.5324 | 0.3158 | 0.4694 | 0.4692 | 0.6690 | -0.4947 | -0.4448 | -0.5779 | -0.4904 | -0.6386 | -0.6410 | 0.4869 | -0.5990 |
| g__Facklamia | -0.4289 | -0.5536 | 0.6891 | 0.5698 | 0.4098 | 0.5615 | 0.6152 | -0.4679 | -0.4991 | -0.4999 | -0.4523 | -0.4382 | -0.6238 | 0.5861 | 0.4991 | 0.4445 | 0.6784 | 0.3671 | 0.5069 | -0.5381 | 0.4835 |
| g__Faecalibaculum | 0.5455 | 0.5071 | -0.6857 | -0.5074 | -0.6785 | -0.8252 | -0.5232 | 0.4406 | 0.4895 | 0.5779 | 0.4825 | 0.5123 | 0.6783 | -0.5679 | -0.5385 | -0.6084 | -0.5385 | -0.6760 | -0.5105 | 0.4825 | -0.5874 |
| g__Romboutsia | 0.7622 | 0.7324 | -0.6937 | -0.7341 | -0.3004 | -0.1469 | -0.7190 | 0.6993 | 0.8042 | 0.5359 | 0.6294 | 0.7544 | 0.7133 | -0.6455 | -0.7552 | -0.5035 | -0.7203 | -0.5569 | -0.6084 | 0.6923 | -0.6224 |
| g__Staphylococcus | -0.5315 | -0.5317 | 0.7495 | 0.5938 | 0.5866 | 0.9231 | 0.5232 | -0.5874 | -0.5734 | -0.5114 | -0.4755 | -0.4912 | -0.7063 | 0.5609 | 0.4615 | 0.5804 | 0.6084 | 0.5604 | 0.5944 | -0.5385 | 0.5455 |
| g__Turicibacter | 0.8881 | 0.8028 | -0.6897 | -0.8169 | -0.5371 | -0.3916 | -0.7474 | 0.8671 | 0.9091 | 0.4553 | 0.7552 | 0.8000 | 0.8182 | -0.7831 | -0.7203 | -0.7413 | -0.7692 | -0.7846 | -0.7483 | 0.7972 | -0.7832 |

| **P value** | | | | | | | | | | | | | | | | | | | | | |
| --- | --- | --- | --- | --- | --- | --- | --- | --- | --- | --- | --- | --- | --- | --- | --- | --- | --- | --- | --- | --- | --- |
| **Name** | **Traits of cerebral injury** | | | | | | | | | | **Traits of intestinal barrier** | | | | | | | **Factors of thrombosis risk** | | | |
|  | **Relative body weight** | **Number of Nissl possitive cell** | **Longa Score** | **mNSS Score** | **S100B in serum** | **NGB in serum** | **Cerebral infarction volume** | **Claudin-1 in brain** | **Occludin in brain** | **ZO-1 in brain** | **Claudin-1 in colon** | **Occludin in colon** | **ZO-1 in colon** | **FD4 in plasma** | **DAO in serum** | **LPS in serum** | **D-Lactate in serum** | **T-CHO** | **TG** | **HDL** | **LDL** |
| g__Adlercreutzia | 0.7162 | 0.5317 | 0.2873 | 0.9823 | 0.3922 | 0.0301 | 0.7079 | 0.5883 | 0.6672 | 0.2906 | 0.5883 | 0.7863 | 0.4991 | 0.3867 | 0.8690 | 0.6672 | 0.5883 | 0.8372 | 0.8517 | 0.9212 | 0.8002 |
| g__Brachybacterium | 0.0489 | 0.0247 | 0.0271 | 0.0181 | 0.0102 | 0.1691 | 0.0089 | 0.0774 | 0.0694 | 0.0961 | 0.0181 | 0.0645 | 0.0145 | 0.0041 | 0.0107 | 0.0145 | 0.0043 | 0.0244 | 0.0530 | 0.0378 | 0.0073 |
| g__Collinsella | 0.0324 | 0.0208 | 0.0638 | 0.0595 | 0.0069 | 0.0280 | 0.0447 | 0.0204 | 0.0301 | 0.1858 | 0.0240 | 0.1137 | 0.0666 | 0.0062 | 0.1542 | 0.0204 | 0.0301 | 0.0341 | 0.0839 | 0.0400 | 0.0458 |
| g__Corynebacterium | 0.3309 | 0.1449 | 0.0384 | 0.1939 | 0.0500 | 0.0002 | 0.1401 | 0.2010 | 0.3425 | 0.0999 | 0.1926 | 0.2386 | 0.1041 | 0.1115 | 0.2367 | 0.1338 | 0.2463 | 0.0703 | 0.1614 | 0.2274 | 0.1041 |
| g__Dubosiella | 0.0660 | 0.1824 | 0.0466 | 0.0695 | 0.1192 | 0.0034 | 0.1501 | 0.1027 | 0.0748 | 0.3174 | 0.1237 | 0.1238 | 0.0174 | 0.1020 | 0.1473 | 0.0490 | 0.1055 | 0.0254 | 0.0247 | 0.1084 | 0.0396 |
| g__Facklamia | 0.1642 | 0.0618 | 0.0132 | 0.0531 | 0.1858 | 0.0575 | 0.0332 | 0.1251 | 0.0986 | 0.0979 | 0.1399 | 0.1542 | 0.0302 | 0.0452 | 0.0986 | 0.1477 | 0.0153 | 0.2404 | 0.0926 | 0.0711 | 0.1113 |
| g__Faecalibaculum | 0.0707 | 0.0925 | 0.0138 | 0.0922 | 0.0153 | 0.0017 | 0.0809 | 0.1542 | 0.1096 | 0.0490 | 0.1154 | 0.0886 | 0.0188 | 0.0541 | 0.0749 | 0.0400 | 0.0749 | 0.0158 | 0.0936 | 0.1154 | 0.0488 |
| g__Romboutsia | 0.0059 | 0.0068 | 0.0123 | 0.0066 | 0.3428 | 0.6511 | 0.0084 | 0.0145 | 0.0027 | 0.0725 | 0.0324 | 0.0046 | 0.0121 | 0.0234 | 0.0066 | 0.0988 | 0.0110 | 0.0600 | 0.0400 | 0.0159 | 0.0348 |
| g__Staphylococcus | 0.0793 | 0.0752 | 0.0050 | 0.0418 | 0.0450 | 0.0000 | 0.0809 | 0.0488 | 0.0555 | 0.0893 | 0.1213 | 0.1048 | 0.0133 | 0.0578 | 0.1338 | 0.0521 | 0.0400 | 0.0581 | 0.0458 | 0.0749 | 0.0707 |
| g__Turicibacter | 0.0001 | 0.0017 | 0.0131 | 0.0012 | 0.0717 | 0.2096 | 0.0052 | 0.0004 | 0.0000 | 0.1369 | 0.0066 | 0.0018 | 0.0020 | 0.0026 | 0.0110 | 0.0082 | 0.0053 | 0.0025 | 0.0074 | 0.0032 | 0.0041 |

Table S6. The final altered metabolites

| Metabolites | FC | raw.p | VIP |
| --- | --- | --- | --- |
| (-)-Wikstromol | 6.6776 | 0.047628 | 1.533787798 |
| (22S)-Acetoxy-3alpha,15alpha-dihydroxylanosta-7,9(11),24-trien-26-oic acid | 603.78 | 0.03209 | 1.518182702 |
| 3'-Deoxyoleacein | 1.7271 | 0.0081527 | 1.861173052 |
| 5alpha-Hydroxytriptolide | 2.075 | 0.036016 | 1.621603969 |
| Amygdalin | 3.2192 | 0.02838 | 1.60571576 |
| Chelidonine | 2.082 | 0.039486 | 1.505007349 |
| Chrysophanein | 76.757 | 0.0022007 | 2.011332413 |
| Daidzin | 1068.6 | 0.013988 | 1.892250719 |
| Diferuloylputrescine | 2.389 | 0.035762 | 1.545794067 |
| Echinocystic acid | 2.0486 | 0.018843 | 1.70945644 |
| Fasciculol C | 7.5742 | 0.034993 | 1.688862375 |
| Fenchyl acetate | 65.581 | 0.022524 | 1.659680375 |
| Glycyrrhetinic Acid | 20.292 | 0.0021727 | 2.116792548 |
| Guanosine | 2.2165 | 0.044003 | 1.42739919 |
| Hydratopyrrhoxanthinol | 3.279 | 0.0073256 | 1.938149612 |
| Indolelactic acid | 9.3317 | 0.031755 | 1.520035802 |
| INDOPHENOL | 6.1723 | 0.046728 | 1.428404713 |
| Jacarandic acid | 3.3026 | 0.020819 | 1.819168729 |
| Lexacalcitol | 23.282 | 0.0099717 | 1.969828316 |
| LysoPC(18:3(6Z,9Z,12Z)/0:0) | 2.2331 | 0.025609 | 1.636850851 |
| LysoPE(0:0/16:0) | 2.3169 | 0.020319 | 1.527202779 |
| LysoPE(16:0/0:0)_1 | 1.7942 | 0.031828 | 1.391723412 |
| LysoPE(16:0/0:0)_2 | 1.8595 | 0.020703 | 1.517933972 |
| Manzamine A | 3.1593 | 0.010408 | 1.890996506 |
| MG(18:1(9Z)-O(12,13)/0:0/0:0) | 2.0841 | 0.037412 | 1.423139821 |
| Neogitogenin | 2.0816 | 0.011758 | 1.796431529 |
| Oxybenzone | 1.8421 | 0.041137 | 1.716130199 |
| Oxymetholone | 2.0831 | 0.008368 | 1.802799242 |
| Polyporusterone F | 3.9521 | 0.0019065 | 1.911931252 |
| Puerarin | 1740.1 | 0.01001 | 1.937583686 |
| Soyasapogenol F | 17.64 | 0.0061289 | 2.002035357 |
| Uridine | 3.8827 | 0.018147 | 1.660881719 |
| Vitexin | 43.884 | 0.0074202 | 1.999992893 |
| (-)-Norephedrine | 1.7033 | 0.15753 | 1.111652289 |
| (+/-)9-HpODE | 1.6451 | 0.050125 | 1.407958909 |
| (3beta,17alpha,23S)-17,23-Epoxy-3,29-dihydroxy-27-norlanosta-7,9(11)-diene-15,24-dione | 1.4717 | 0.10608 | 1.209382208 |
| [16]-Gingerol | 20.18 | 0.21885 | 1.181594143 |
| 1-[7-(dimethylamino)-3,5-dihydro-2H-1,4-benzoxazepin-4-yl]-3-methoxypropan-1-one | 1.9697 | 0.13155 | 1.251468266 |
| 10-Hydroxy-8-nor-2-fenchanone glucoside | 1.8869 | 0.21827 | 1.004548797 |
| 17-Hydroxymethylethisterone | 3.027 | 0.051337 | 1.339692168 |
| 2,3-dinor Prostaglandin E1 | 1.2912 | 0.10007 | 1.349863561 |
| 2',4'-Dihydroxyacetophenone | 7.8098 | 0.099563 | 1.32640814 |
| 2-Hydroxy-3-Methylbutyric Acid | 5.2999 | 0.058671 | 1.440811519 |
| 3beta-Hydroxy-17-(1H-imidazol-1-yl)androsta-5,16-diene | 1.1253 | 0.12703 | 1.079960007 |
| 4-Hydroxyquinoline | 1.3833 | 0.17993 | 1.219811876 |
| 4'-O-Methyl-(-)-epicatechin-7-O-sulphate | 75.911 | 0.062963 | 1.586794268 |
| 5-(2'-Carboxyethyl)-4,6-Dihydroxypicolinate | 18.208 | 0.078531 | 1.407338584 |
| 7,4'-Dihydroxyflavone | 2.5976 | 0.11568 | 1.199740753 |
| 7-Amino-4-hydroxy-2-naphthalenesulfonic acid | 1.6336 | 0.1014 | 1.265901728 |
| 7-Deoxydoxorubicinone | 2.1009 | 0.14828 | 1.09003037 |
| 9-Hydroxylinoleic acid | 1.3991 | 0.12829 | 1.201631064 |
| Acacetin | 11.258 | 0.10527 | 1.335442644 |
| Artemorin | 1.5623 | 0.11034 | 1.261517771 |
| Betaine | 1.5828 | 0.12093 | 1.184030464 |
| Bisnoryangonin | 59.492 | 0.11236 | 1.406857005 |
| Capecitabine | 1.4325 | 0.14931 | 1.205737784 |
| Carboprost | 7.8682 | 0.17814 | 1.093950565 |
| Carvedilol | 102.27 | 0.092529 | 1.355956993 |
| Catechin 7-glucoside | 7.9461 | 0.1423 | 1.172757271 |
| Cefroxadine | 3.5545 | 0.20831 | 1.007945392 |
| Chrysoeriol | 62.108 | 0.16782 | 1.185093489 |
| Coclaurine | 5.6212 | 0.16622 | 1.138233156 |
| Coumestrol | 1.2913 | 0.18835 | 1.192646896 |
| Cynaropicrin | 29.05 | 0.2368 | 1.038072165 |
| Daidzein | 3.0475 | 0.13777 | 1.145886571 |
| Dehydrosoyasaponin I | 32.931 | 0.061055 | 1.414608806 |
| Deoxydihydro-artemisinin | 1.6025 | 0.18473 | 1.099622043 |
| Dextran | 2.1879 | 0.17651 | 1.030795161 |
| Dihydrogenistin | 2.6342 | 0.11407 | 1.393126972 |
| Dihydroxylysinonorleucine | 2.1073 | 0.095758 | 1.2210906 |
| Domoic acid | 233.3 | 0.10687 | 1.23011647 |
| Enol-phenylpyruvate | 6.3529 | 0.1318 | 1.129152542 |
| Ferulic Acid | 1.5468 | 0.19951 | 1.022960529 |
| Formononetin | 41.29 | 0.078155 | 1.425027575 |
| Galactosylhydroxylysine | 2.0856 | 0.16487 | 1.062951723 |
| Gentiobiose | 2.2043 | 0.12297 | 1.149441432 |
| Gentisic Acid | 2.6401 | 0.055254 | 1.489940204 |
| Gibberellin A3 | 2.4768 | 0.1464 | 1.083795167 |
| Gibberellin A8 | 2.4224 | 0.14365 | 1.013167908 |
| Ginsenoside C-K | 1308.2 | 0.10695 | 1.321753657 |
| Ginsenoside F1 | 66.287 | 0.11717 | 1.274527634 |
| Glutaminylphenylalanine | 10.892 | 0.05104 | 1.369952104 |
| Glycitein | 15.975 | 0.098999 | 1.340159978 |
| Guanine | 1.7528 | 0.19556 | 1.052510195 |
| HDMBOA-Glc | 25.016 | 0.09044 | 1.351252771 |
| Indolapril | 1.6833 | 0.14685 | 1.203404884 |
| Islatravir | 33.543 | 0.12322 | 1.224402967 |
| Isoferulic Acid | 1.7893 | 0.15024 | 1.102186792 |
| Lenticin | 16.71 | 0.095593 | 1.193045719 |
| LysoPA(0:0/18:1(9Z)) | 2.1773 | 0.1421 | 1.080060928 |
| LysoPC(0:0/18:2(9Z,12Z)) | 1.7228 | 0.060548 | 1.396344647 |
| LysoPE(0:0/18:2(9Z,12Z)) | 1.5114 | 0.13202 | 1.160536168 |
| LysoPE(18:2/0:0) | 1.7455 | 0.064284 | 1.399275223 |
| LysoPI(18:2(9Z,12Z)/0:0) | 2.5348 | 0.089347 | 1.271158829 |
| Maltotriose | 2.192 | 0.13216 | 1.151950715 |
| Monensin | 10.349 | 0.065576 | 1.418472865 |
| Mytilin B | 6.3442 | 0.1616 | 1.146848675 |
| N-(1,3-Dihydroxyoctadec-4-en-2-yl)acetamide | 1.4894 | 0.11992 | 1.336289826 |
| N-Desmethylaminopyrine | 12.525 | 0.12171 | 1.267109032 |
| N-Docosahexaenoyl Aspartic acid | 44.507 | 0.21336 | 1.211113427 |
| Osthenol | 120.65 | 0.11431 | 1.408233682 |
| Ovalicin | 1.3879 | 0.068171 | 1.442109694 |
| Pemoline | 2.3908 | 0.070554 | 1.50270117 |
| PG(PGE1/18:1(9Z)) | 13.653 | 0.2543 | 1.018174252 |
| PGP(PGF1alpha/22:4(7Z,10Z,13Z,16Z)) | 9.3251 | 0.18762 | 1.163832204 |
| Phaseic acid | 29.754 | 0.12213 | 1.071571817 |
| Phloretin | 6.7668 | 0.14653 | 1.271052755 |
| Plumieride | 13.5 | 0.21564 | 1.006393912 |
| Protocatechuic Acid | 2.2865 | 0.080779 | 1.38646329 |
| Ptaquiloside | 1.4516 | 0.09967 | 1.248633858 |
| Rutarin | 5.2601 | 0.066982 | 1.343696281 |
| Sakuranetin | 226.4 | 0.10715 | 1.33456113 |
| Salbutamol | 1.7637 | 0.064461 | 1.387734363 |
| Ser-Leu | 1.5597 | 0.18615 | 1.103345873 |
| Simmondsin | 2.7 | 0.20778 | 1.008724443 |
| Soyasapogenol B 3-O-[a-rhamnosyl-(1->4)-b-D-galactosyl-(1->4)-b-D-glucuronide] | 17.086 | 0.072155 | 1.360744488 |
| Soyasapogenol B 3-O-b-D-glucuronide | 13.057 | 0.12243 | 1.18442044 |
| Soyasaponin A1 | 4.8171 | 0.19006 | 1.036789139 |
| Soyasaponin I | 21.346 | 0.11428 | 1.2171718 |
| Soybean saponin BG | 85.174 | 0.083131 | 1.319584823 |
| Trans-Piceid | 4.829 | 0.13407 | 1.193664746 |
| Tricin | 2.6867 | 0.063218 | 1.408411731 |
| Valganciclovir, (S)- | 1.8913 | 0.14665 | 1.080082099 |
| Vignafuran | 2.7318 | 0.12036 | 1.345772355 |
| VIPROSTOL | 167.23 | 0.075863 | 1.242524495 |
| (3beta,5alpha,6alpha,7beta,14alpha,22E,24R)-5,6-Epoxyergosta-8,22-diene-3,7,14-triol | 0.3981 | 0.014174 | 1.749018545 |
| 12-Oxo-2,3-dinor-10,15-phytodienoic acid | 0.36233 | 0.032209 | 1.61609785 |
| 13(S)-Hydroperoxylinolenic acid | 0.43614 | 0.048985 | 1.549320427 |
| 1-a,24R,25-Trihydroxyvitamin D2 | 0.50626 | 0.030957 | 1.55899778 |
| 2,3-Secoporrigenin | 0.19155 | 0.011117 | 1.849712573 |
| 27-Deoxy-5b-cyprinol | 0.42174 | 0.0072009 | 1.889032349 |
| Acetylshikonin | 0.46327 | 0.028625 | 1.697689629 |
| Artemisin | 0.085755 | 0.036366 | 1.570632536 |
| Buprenorphine | 0.56063 | 0.028231 | 1.636051028 |
| Chenodeoxycholic Acid | 0.15444 | 0.042481 | 1.55386269 |
| Deoxycholic Acid | 0.3451 | 0.032119 | 1.643839298 |
| Desglucocoroloside | 0.41525 | 0.034595 | 1.520270457 |
| Hexylresorcinol | 0.21928 | 0.045091 | 1.619871168 |
| Isoursodeoxycholic acid | 0.17246 | 0.0014025 | 2.09256307 |
| Ixabepilone | 0.48088 | 0.0073758 | 1.941072917 |
| L-Alaninamide, N-acetyl-L-tyrosyl-L-valyl-N-((1S)-2-carboxy-1-formylethyl)- | 0.22671 | 0.014862 | 1.791949401 |
| L-beta-aspartyl-L-threonine | 0.158 | 0.022979 | 1.613996078 |
| Monoketocholic acid | 0.40125 | 0.0217 | 1.738328863 |
| N6-Methyl-2'-deoxyadenosine | 0.5187 | 0.0014365 | 2.070965558 |
| N-Acetyl-Glutamic Acid | 0.4428 | 0.049272 | 1.464342115 |
| N-Acetylmuramate | 0.27762 | 0.046587 | 1.505051034 |
| Sambacin | 0.21209 | 0.027815 | 1.638869745 |

Table S7. Pathway analysis between the MCAO group and BHTD-H group.

| **Pathway Name** | **p** | **-log(p)** | **Holm p** | **FDR** | **Impact** | **Match Metabolites** |
| --- | --- | --- | --- | --- | --- | --- |
| [Glycerophospholipid metabolism](https://genap.metaboanalyst.ca/MetaboAnalyst/Secure/pathway/PathResultView.xhtml) | 0.20998 | 0.67783 | 1 | 1 | 0.13987 | LysoPA  (0:0/18:1(9Z)) |
| [Glycine, serine and threonine metabolism](https://genap.metaboanalyst.ca/MetaboAnalyst/Secure/pathway/PathResultView.xhtml) | 0.19413 | 0.7119 | 1 | 1 | 0.05034 | Betaine |
| [Primary bile acid biosynthesis](https://genap.metaboanalyst.ca/MetaboAnalyst/Secure/pathway/PathResultView.xhtml) | 0.033327 | 1.4772 | 1 | 1 | 0.03595 | 27-Deoxy-5b-cyprinol  Chenodeoxycholic Acid |
| [Tryptophan metabolism](https://genap.metaboanalyst.ca/MetaboAnalyst/Secure/pathway/PathResultView.xhtml) | 0.23576 | 0.62754 | 1 | 1 | 0.0139 | Indole lactic acid |
| [Purine metabolism](https://genap.metaboanalyst.ca/MetaboAnalyst/Secure/pathway/PathResultView.xhtml) | 0.062718 | 1.2026 | 1 | 1 | 0.01281 | Guanosine Guanine |
| [Glycerolipid metabolism](https://genap.metaboanalyst.ca/MetaboAnalyst/Secure/pathway/PathResultView.xhtml) | 0.098834 | 1.0051 | 1 | 1 | 0.01246 | LysoPA  (0:0/18:1(9Z)) |
| [Phosphatidylinositol signaling system](https://genap.metaboanalyst.ca/MetaboAnalyst/Secure/pathway/PathResultView.xhtml) | 0.16709 | 0.77704 | 1 | 1 | 0.00152 | LysoPA  (0:0/18:1(9Z)) |
| [Phenylalanine metabolism](https://genap.metaboanalyst.ca/MetaboAnalyst/Secure/pathway/PathResultView.xhtml) | 0.062852 | 1.2017 | 1 | 1 | 0 | Enol-phenylpyruvate |
| [Arginine biosynthesis](https://genap.metaboanalyst.ca/MetaboAnalyst/Secure/pathway/PathResultView.xhtml) | 0.086981 | 1.0606 | 1 | 1 | 0 | N-Acetyl-Glutamic Acid |
| [Tyrosine metabolism](https://genap.metaboanalyst.ca/MetaboAnalyst/Secure/pathway/PathResultView.xhtml) | 0.24082 | 0.6183 | 1 | 1 | 0 | Gentisic Acid |

**Match Status** refers to the number of differential metabolites matching the signal pathway. **p** refers to the original *p* value calculated from the enrichment analysis. **Holm p** refers to the *p* value adjusted by Holm-Bonferroni method. **FDR** refers to the *p* value adjusted using False Discovery Rate. **Impact** refers to the pathway impact value calculated from pathway topology analysis.

**
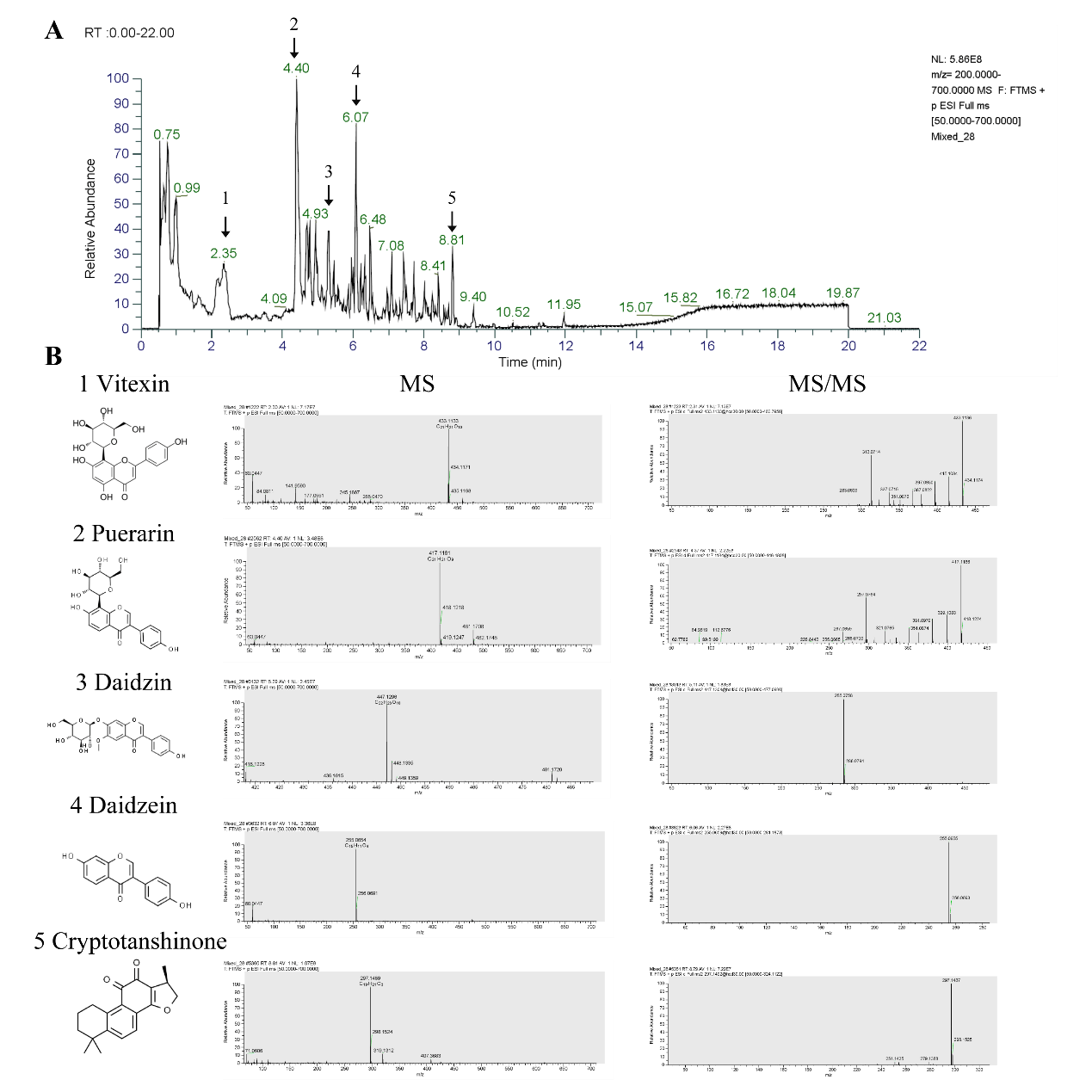
**

**Figure S1. LC-MS/MS spectrums of BHTD.** (A) The representative total ion current chromatograms (TIC) in ESI positive. (B) MS and MS/MS spectrums of five main chemical components.

**
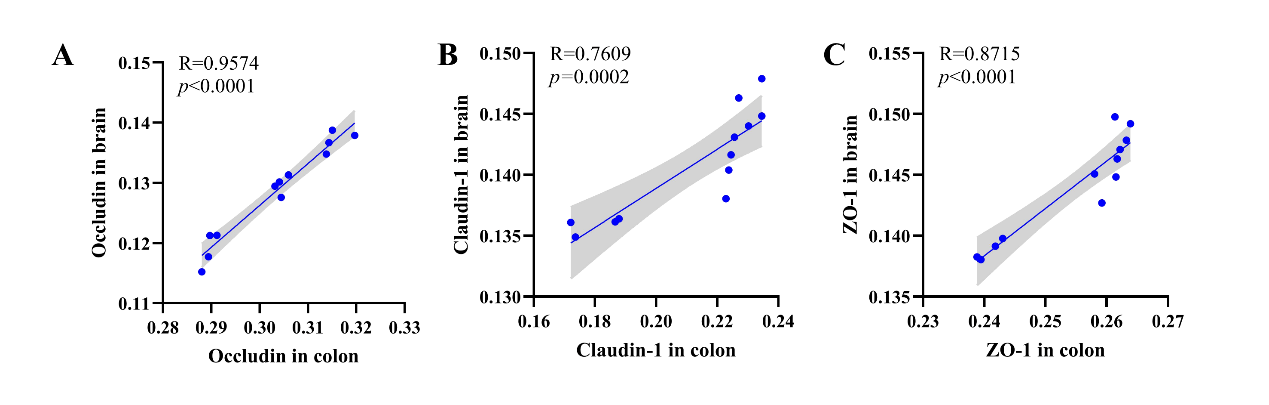
**

**Figure S2. Pearson correlation analyses of the levels of Occludin (A), Claudin-1 (B) and ZO-1 (C) between the brain and colon.**

**
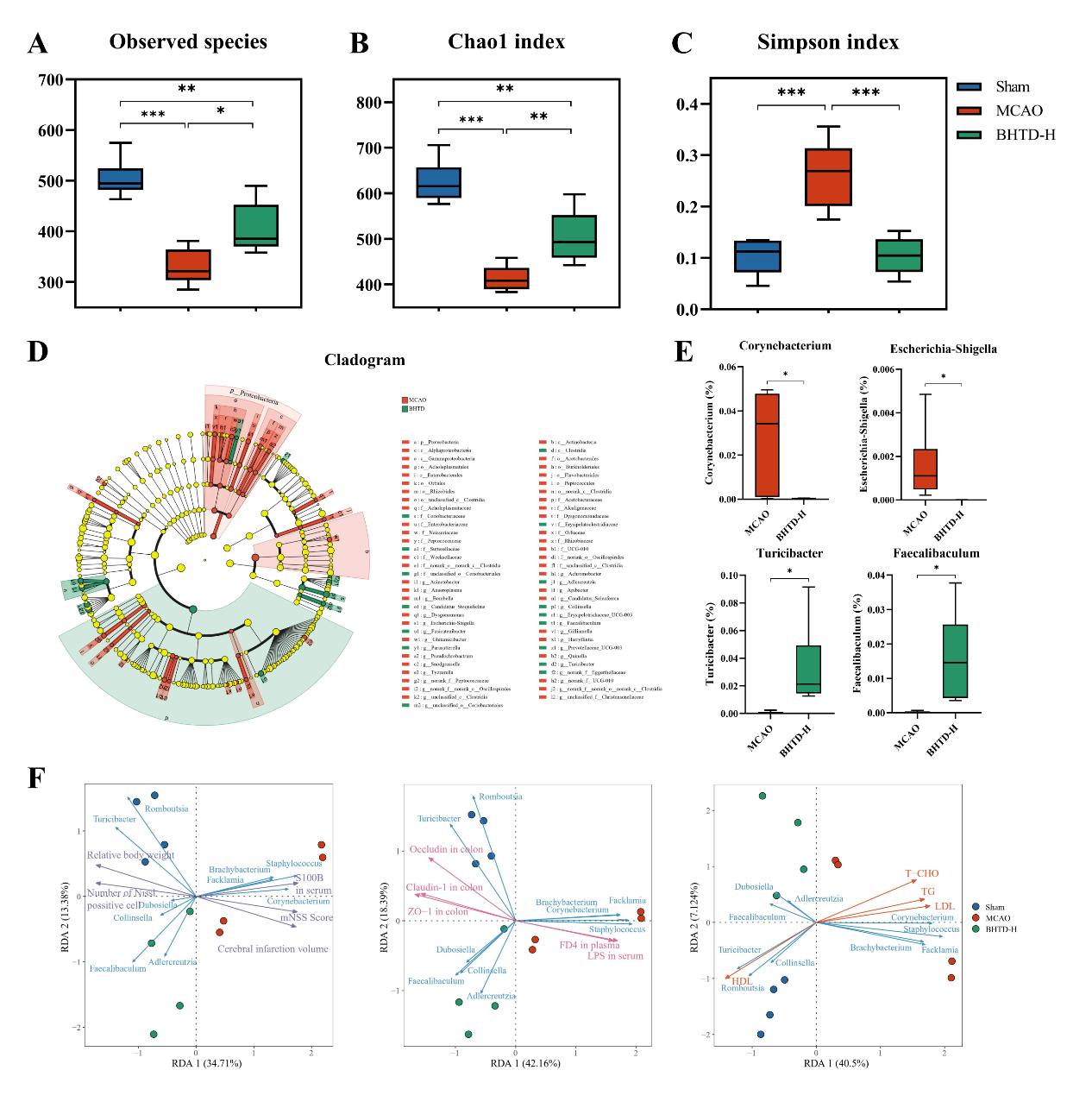
**

**Figure S3. The effect of BHTD supplementation on gut microbiota.** (A-C) α-Diversity analysis including the observed species, Chao1 and Simpson indexes. (Students’t test, n = 6) (D) The cladogram of characteristic genus. (E) The relative of absolute abundance of *Corynebacterium*, *Escherichia-Shigella*, *Turicibacter* and *Faecelibaculum*. (n=6) (F) RDA analysis between the gut microbiota abundance and efficacy parameters, including traits of cerebral injury and intestinal barrier and risk factors for thrombosis. (n=4)

**
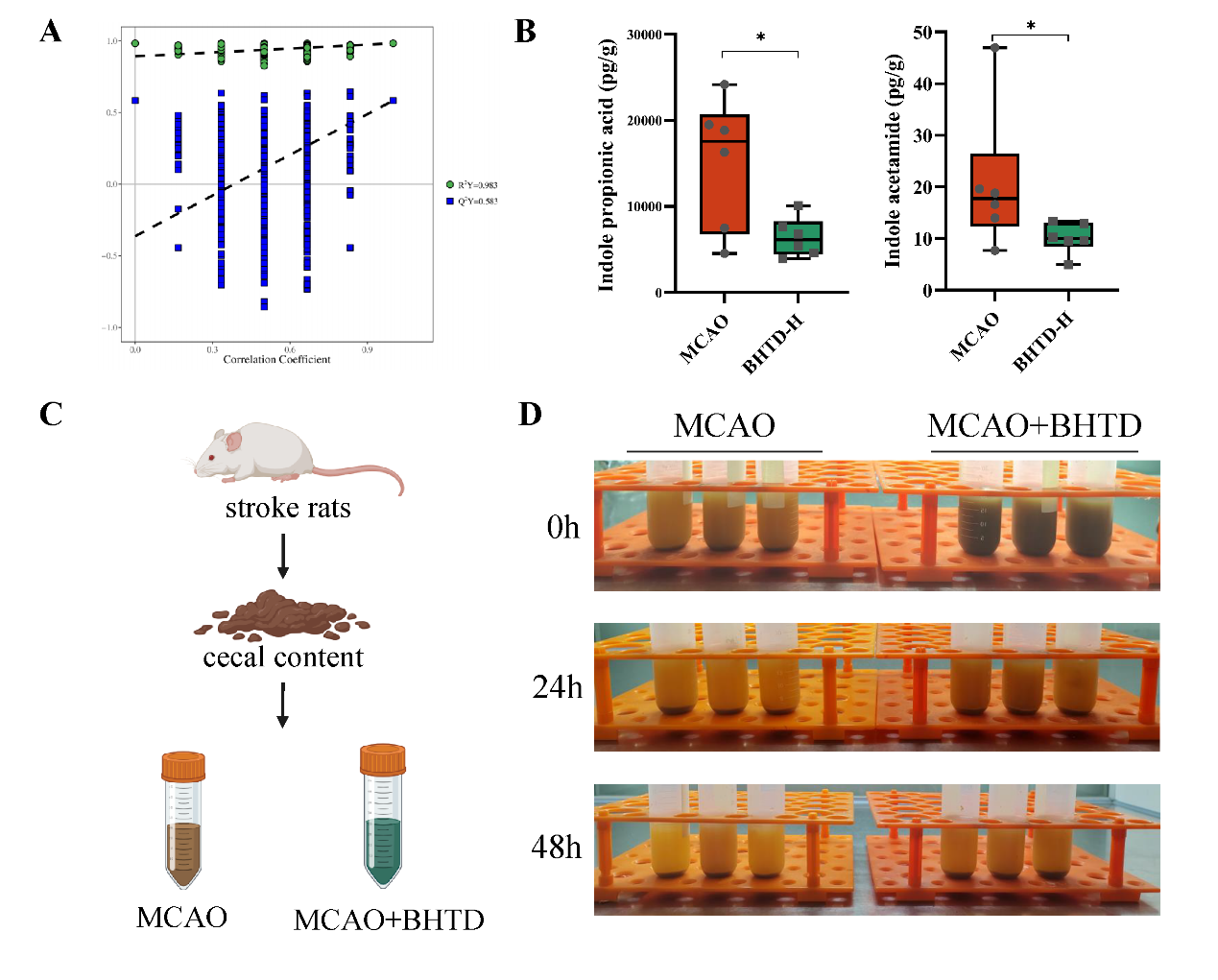
**

**Figure S4. BHTD altered the gut microbial profiles and promoted the production of gut microbiota-derived indole lactic acid.** (A) Permutation test based on the OPLS-DA model. (B) Differential indoles detected by targeted metabolomics. (C) Schematic diagram of in vitro fermentation experiment. (D) Photographs were taken of in vitro fermentation experiment at 0th, 24th and 48th hours.
